# Alignment of Rutaceae Genomes Reveals Lower Genome Fractionation Level Than Eudicot Genomes Affected by Extra Polyploidization

**DOI:** 10.3389/fpls.2019.00986

**Published:** 2019-08-06

**Authors:** Jiaqing Yuan, Jinpeng Wang, Jigao Yu, Fanbo Meng, Yuhao Zhao, Jing Li, Pengchuan Sun, Sangrong Sun, Zhikang Zhang, Chao Liu, Chendan Wei, He Guo, Xinyu Li, Xueqian Duan, Shaoqi Shen, Yangqin Xie, Yue Hou, Jin Zhang, Tariq Shehzad, Xiyin Wang

**Affiliations:** ^1^School of Life Sciences, North China University of Science and Technology, Tangshan, China; ^2^Center for Genomics and Computational Biology, North China University of Science and Technology, Tangshan, China; ^3^Plant Genome Mapping Laboratory, University of Georgia, Athens, GA, United States

**Keywords:** rutaceae, whole-genome duplication, genome alignment, gene collinearity, duplicated genes, genome fractionation

## Abstract

Owing to their nutritional and commercial values, the genomes of several citrus plants have been sequenced, and the genome of one close relative in the Rutaceae family, atalantia (*Atalantia buxifolia*), has also been sequenced. Here, we show a family-level comparative analysis of Rutaceae genomes. By using grape as the outgroup and checking cross-genome gene collinearity, we systematically performed a hierarchical and event-related alignment of Rutaceae genomes, and produced a gene list defining homologous regions based on ancestral polyploidization or speciation. We characterized genome fractionation resulting from gene loss or relocation, and found that erosion of gene collinearity could largely be described by a geometric distribution. Moreover, we found that well-assembled Rutaceae genomes retained significantly more genes (65–82%) than other eudicots affected by recursive polyploidization. Additionally, we showed divergent evolutionary rates among Rutaceae plants, with sweet orange evolving faster than others, and by performing evolutionary rate correction, re-dated major evolutionary events during their evolution. We deduced that the divergence between the Rutaceae family and grape occurred about 81.15–91.74 million years ago (mya), while the split between citrus and atalantia plants occurred <10 mya. In addition, we showed that polyploidization led to a copy number expansion of key gene families contributing to the biosynthesis of vitamin C. Overall, the present effort provides an important comparative genomics resource and lays a foundation to understand the evolution and functional innovation of Rutaceae genomes.

## Introduction

The genus *Citrus L*., belonging to the subfamily Aurantioideae in the family Rutaceae, comprises many important fruit-producing plants. Citrus is grown in at least 114 countries (Talon and Gmitter, 2008; Liu et al., 2012), and widely cultivated citrus species include sweet orange [*C. sinensis* (L.) Osbeck], mandarin clementine (*C. clementina* hort. ex Tanaka), pummelo (*C. grandis* Osbeck or *C. maxima* Merr.), grapefruit (*Citrus paradisi* Macf.), lemon [*Citrus limon* (L.) Burm. f.], and papeda (*Citrus ichangensis* Swingle) (Barrett and Rhodes, 1976; Moore, 2001). With a long cultivated history, citrus plays important roles for its values from daily life to commercial activity, providing rich vitamins, antioxidant compounds for healthy diets and various flavorings such as beverages (Cheong et al., 2012; Liu et al., 2012; Singh et al., 2014).

Citrus has undergone a complicated history of inbreeding and artificial breeding (Scora, 1975; Barrett and Rhodes, 1976; Nicolosi et al., 2000; Wu et al., 2014, 2018; Curk et al., 2016). Mainly for their economic importance, genome sequences of citrus plants and a close Rutaceae relative have been deciphered, including sweet orange, clementine, pummelo, papeda, citron, and atalantia (Xu et al., 2013; Wu et al., 2014; Wang et al., 2017b). The high-quality clementine genome sequence produced by the International Citrus Genome Consortium (ICGC: http://www.citrus.genome.ucr.edu/) includes 301.4 Mb with 1,398 scaffolds and L50 reached to 31.4 MB (Wu et al., 2014). The draft genome of sweet orange was released with total contig length about 320.5 Mb and 4,811 scaffolds (>500 b p) (Xu et al., 2013). The other 4 genomes were released by the same research group, including a highest-quality assembled pummelo genome with total scaffold length of about 301.95 Mb positioned to 117 scaffolds, and draft genomes of papeda, citron, and atalantia, which were not assembled to the chromosomal level yet (Wang et al., 2017b).

Polyploidization is widespread during the evolution of land plants (Soltis and Soltis, 1999; Adams and Wendel, 2005; Maere et al., 2005; Soltis et al., 2009). A core-eudicot-common hexaploidy (ECH) (Bowers et al., 2003) was revealed from the sequence of the Arabidopsis genome (The Arabidopsis Genome Initiative, 2000), and clearly deciphered with the availability of the grape genome (Jaillon et al., 2007). The ECH was also described with citrus genomes (Xu et al., 2013), tripling all chromosomes and all genes in their common ancestral genome (Soltis and Soltis, 1999; Aury et al., 2006; Soltis et al., 2009; Wang et al., 2018). After the ECH, ancestral genomes experienced extensive gene loss, genomic fractionation, and chromosomal rearrangement, most likely before the split of eudicot plants and continuing at much lower levels in each new species (Wang et al., 2005; Freeling et al., 2008; Buggs et al., 2012; Sankoff and Zheng, 2012). The extant Rutaceae genomes formed with at least 10 chromosome fusions and breakages after the ECH, in each of which thousands of ECH-duplicates or paralogous genes were preserved (Xu et al., 2013). These paralogous genes in Rutaceae plants often have their orthologs preserved, and non-orthologous paralogs from different plants are specifically called outparalogs.

**Figure 1:**
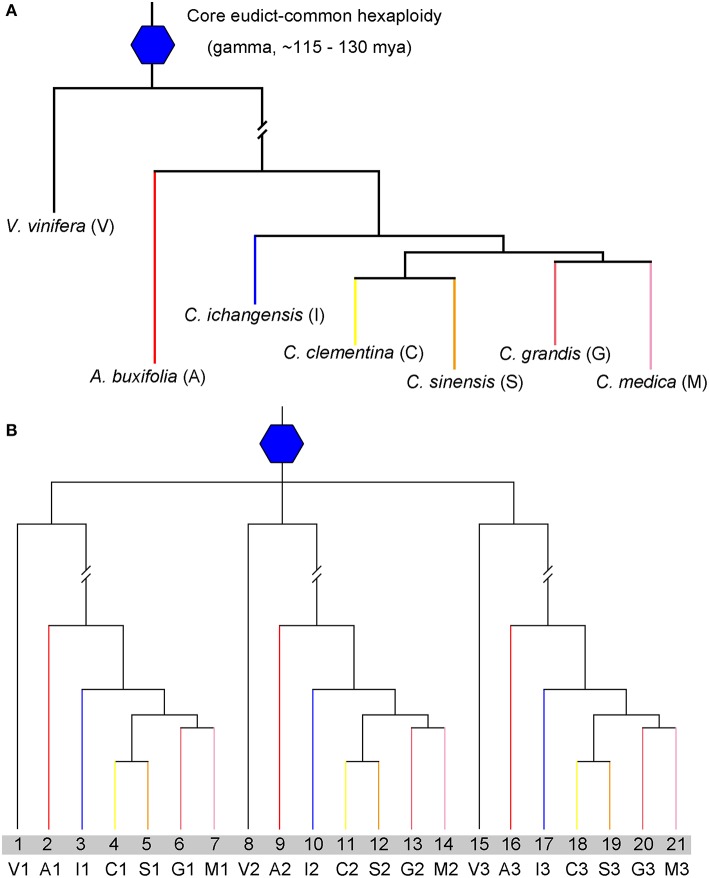
Species and gene phylogenetic tree. **(A)** Phylogenetic tree of *Vitis vinifera* (V), *Atalantia buxifolia* (A), *Citrus ichangensis* (I), *Citrus medica* (M), *Citrus grandis* (G), *Citrus clementine* (C), and *Citrus sinensis* (S). The core eudicot-common hexaploidy is represented by a blue hexagon. **(B)** Gene tree to show paralogs within each genome, V1,V2, and V3 produced by the ECH, also happened in citrus genomes.

The availability of citrus and related genomes provides valuable opportunities to understand their biology and evolution. However, a cross-genome characterization and comparison of genome fractionation, e.g., gene retention and loss, shared orthology among Rutaceae plants after the ECH or after their split, has not been available. Here, we aim at inferring gene collinearity within and between genomes of five citrus and one Rutaceae relative with grape as the outgroup reference, constructing intra- and inter-genomic homology due to polyploidization and speciation, and producing lists of orthologs, paralogs and outparalogs (Figure 1). Moreover, by exploring the above genome alignment, we can deduce pan-family genome fractionation. Further, we can deduce how polyploidization affected copy number variation of gene families, exemplified by vitamin C genes. Overall, the present effort provides an important comparative genomics resource for further biological exploration in Rutaceae and beyond.

## Materials and Methods

### Genetic Material

Citrus and grape genome data and their gene annotations were downloaded from the websites respectively, displayed in Supplementary Table 1.

### Synonymous Nucleotide Substitutions

Synonymous nucleotide substitutions at synonymous sites (Ks) were estimated by using the Nei-Gojobori approach (Nei and Gojobori, 1986) implemented in the Bioperl Statistical module.

### Genomic Homology

BLASTP 2.7.1+ (Altschul et al., 1990) was used to find putative homologous genes (E-value <1e-5, a parameter set to control the similarity between two protein datasets). A loose E-value will not jeopardize the analysis of genomic homology in consideration of divergent evolutionary rates among genes. Credible gene homology will be further supported by gene collinearity, as described later. Genome homology dotplots were produced with a custom in-house program using BLASTP 2.7.1+ results as inputs.

With the putative homologous genes as input information, we ran ColinearScan 1.0.1 to infer collinear genes that help reveal homologous blocks within and between genomes (Wang et al., 2006). Based on similarity (Ks and collinearity gene density) between homologous blocks, we found orthologous and paralogous correspondence between different genomes. Orthologous correspondence shares more collinear genes and smaller Ks values than paralogous correspondence. The correspondence information was used to construct collinear gene tables by filling collinear genes inferred. The collinear gene table was used furthermore to infer gene loss, retention, translocation, etc.

### Pseudo-Chromosome Reconstruction

Firstly, we selected the sequences of sweet orange gene to do BLASTN 2.7.1+ searches against scaffolds of citron, papeda, and atalantia, respectively (Altschul et al., 1990). Then, scaffolds were ordered as to their best matched sweet orange genomic segments.

### Kernel Function Analysis of Ks

To exhibit the enrichment of Ks, we performed a kernel function analysis of the Ks distribution of collinear genes within a genome or between genomes. With Ks values assumed to follow a normal distribution, Matlab R2014a was adopted to display the distribution of Ks. Then, a curve fitting tool cftool was used to obtain the proper curves by adjusting related parameters, with the popular Gaussian equation. We adjusted the R-square close to 1, the SSE and RMSE as small as possible and the coefficients with 95% confidence bound, and finally determined the goodness of fit.

### Evolutionary Dating Correction

Assuming a normal distribution of Ks values, the principle curve was used to represent the corresponding evolutionary event. More detailed methods can be found in our previous publications (Wang et al., 2017a, 2018), with the equations, below, modified due to the lack of an additional polyploidization in citrus plants after the ECH. Considering ECH-related Ks peaks of studied species, we adopted grape as the standard for being the slowest in all studied plants, and corrected the evolutionary rates of the Rutaceae plants. The Maximum likelihood estimate μ can be inferred from the Ks average. Supposing a grape duplicated gene pair to have Ks value that is a random variable $X_{G}\sim \left(\mu_{G},{\sigma_{G}}^{2}\right)$, and for a duplicated gene pair in another genome to have Ks of $X_{i}\sim \left(\mu_{i},{\sigma_{i}}^{2}\right)$, we determine the relative difference as:

$$\begin{array}{l} r=\left(\mu_{i}-\mu_{G}\right)/\mu_{G}. \end{array}$$

To get the corrected *X*_*i*−*correction*_ ~ (μ_*i*−*correction*_, ${\sigma^{2}}_{i-correction})$, we defined the correction coefficient as:

$$\begin{array}{l} \frac{\mu_{i-correction}}{\mu_{i}}=\frac{\mu_{G}}{\mu_{i}}=\lambda_{i}, \end{array}$$

and $\mu_{i-correction}=\frac{\mu_{G}}{\mu_{i}}\times \mu_{i}=\frac{1}{1+r}\times \mu_{i}$.

$$\begin{array}{l} \lambda_{i}=\frac{1}{1+r} \end{array}$$

then,

$$\begin{array}{l} X_{i-correction}\sim \left(\lambda_{i}\mu_{i},{\lambda_{i}}^{2}{\sigma_{i}}^{2}\right) \end{array}$$

To calculate Ks of homologous gene pairs between two Rutaceae plants, *i, j*,supposing the Ks distribution is $X_{ij}\sim \left(\mu_{ij},{\sigma_{ij}}^{2}\right)$, we adopted the algebraic mean of the correction coefficients from two plants,

$$\begin{array}{l} \lambda_{ij}=\left(\lambda_{i}+\lambda_{j}\right)/2, \end{array}$$

then,

$$\begin{array}{l} X_{ij-correction}\sim \left(\lambda_{ij}\mu_{ij},{\lambda_{ij}}^{2}{\sigma_{ij}}^{2}\right). \end{array}$$

Specifically, when one plant is grape, for the other plant, i, we have,

$$\begin{array}{l} X_{iG-correction}\sim \left(\lambda_{i}\mu_{iG},{\lambda_{i}}^{2}{\sigma_{iG}}^{2}\right) \end{array}$$

### Multiple Sequence Alignment and Evolutionary Tree Construction

The original family genes involved in the Vitamin C pathway in sweet orange were downloaded (http://citrus.hzau.edu.cn/orange/). With these 101 genes as reference, we inferred homologous genes in the other Rutaceae plants and grape (Supplementary Table 2). Then, we combined BLASTP 2.7.1+ (score ≥ 150 and identity ≥ 50%) and HMMER 3.0 (http://hmmer.org/) to refine the inference. Sequences were aligned using Clustal X version 2.0 (Larkin et al., 2007). Phylogenetic trees of the genes involved in the Vitamin C pathway were constructed by using MEGA 7 (the Neighbor-joining method and the Bootstrap value: 1,000) (Kumar et al., 2016) and IQTREE 1.6.8 (default running with all protein test models and the Bootstrap value: 1,000) (Hoang et al., 2017; Kalyaanamoorthy et al., 2017) was used for large families.

## Results

### Inference of Gene Collinearity Within and Among Genomes

Gene collinearity in extant genomes, describing genes to have preserved ancestral order in ancient genomes, is key to deciphering the complexity of plant genomes and understanding their evolutionary history. Here, we inferred collinear genes within each Rutaceae genome, and between any two of them. Further, we inferred collinear genes in grape, as the outgroup reference, and between grape and each Rutaceae plant. Numbers of homologous blocks containing collinear genes were shown in Supplementary Tables 3, 4. Since citron, papeda and atalantia were assembled only to the scaffold level, we mapped their scaffolds onto sweet orange pseudo-chromosomes to construct their own likely pseudo-chromosomes. These constructed pseudo-chromosomes are helpful in inferring gene collinearity, though they cannot fully reflect the gene content and structure of the actual chromosomes. There are 3,666 to 4,932 collinear genes (Supplementary Table 4) in a genome, forming 2,110 to 2,856 paralogous pairs, inferred from 176 to 307 homologous blocks with 4 or more collinear genes (Supplementary Table 3). The longest homologous block is between pummelo chromosomes Cg5-Cg9, containing 106 gene pairs. For longer blocks with 20 or more genes, fewer collinear genes were inferred in papeda (921 from 5 blocks) and citron (721 from 2 blocks) possibly due to poor assembly (Supplementary Tables 3, 4). Comparatively, grape has 176 blocks (including 2,116 gene pairs formed by 3,666 genes), fewer than those within a Rutaceae genome (Supplementary Tables 3, 4).

The collinearity between genomes (intergenomic) is higher (more conserved) than within a genome (intragenomic), consistent with speciation following genome duplication and showing well-preserved ancestral Rutaceae, even eudicot, genome structure in extant plants. As to blocks with 4 or more genes, sweet orange has 3,792 collinear genes within its genome and shares 10,342–14,120 collinear genes with other citrus genomes (Supplementary Table 4). Even with atalantia and grape, sweet orange shares 12,789 and 9,108 collinear genes, respectively, much more than its intragenomic collinear gene number. The longest block between citrus and grape is located between citrus chromosome 3 and grape chromosome 18, while the longest block appears between atalantia chromosome 5 and grape chromosome 7. Apparently, clementine and pummelo have the best collinearity, involving 15,716 (64.52%) and 15,474 (55.18%) genes, respectively. Notably, the longest homologous block appeared between pummelo and clementine with 1,356 genes. Similarly, sweet orange has the second longest homologous block with clementine, involving 1,328 collinear genes (Supplementary Table 4).

More specific statistics regarding orthologous, paralogous and outparalogous genes, gene pairs and gene blocks were displayed in Supplementary Tables 3, 4.

### Distinguishing Orthology From Outparalogy

Here, we used the grape genome to distinguish orthologous and outparalogous regions between different genomes. Without considering lineage-specific gene losses or duplications, we would expect that a grape gene (or chromosomal region) had one best matched or orthologous Rutaceae gene (chromosomal region), and two secondary or outparalogous genes (chromosomal regions). Specifically, we illustrated the principle with three grape chromosomes (Vv6, Vv8, and Vv13), being ECH-produced homoeologs/paralogs (Jaillon et al., 2007; Jiao et al., 2011; Wang et al., 2017a), to find their respective orthologous and outparalogous regions in the sweet orange (Cs) genome (Supplemental Figure S1). As to gene collinearity and sequence divergence (measured by using synonymous nucleotide substitution rates, or Ks), Vv6 is orthologous to most of Cs8 (including 347 collinear genes with median Ks = 0.92), and complemented by several smaller fragments in Cs1, Cs2, Cs3, Cs6, and Cs9 (including 8, 92, 21, 21, and 40 collinear genes, respectively), while Vv8 and Vv 13 are mainly orthologous to Cs6 (514 collinear genes with Ks = 0.88) and Cs7 (446 collinear genes with Ks = 0.935), respectively complemented by fragments from other grape chromosomes. Non-orthologous alternative correspondence means outparalogy, often having fewer or extensively diverged collinear genes. For example, for outparalogous correspondence, Vv6-Cs3 share only 5 collinear genes and Vv6-Cs5 share 6 collinear genes; though Vv6-Cs6 share 90 collinear genes, far fewer than Vv6-Cs8 (347) and much more diverged (Ks = 0.93). Likewise, Vv8 and Vv13 also had a similar phenomenon, with orthologous regions much better preserved than outparalogous regions.

Eventually, we managed to distinguish orthologous and outparalogous regions between grape and each Rutaceae plant, and similarly we inferred orthologous and outparalogous regions between any two Rutaceae plants.

Table 1 summarizes the number of duplicate genes in relation to the ECH. In grape, 1,289 paralogous gene pairs, involving 2,364 extant genes, were inferred to be produced by the ECH, accounting for 54.53% of all inferred collinear genes. In citrus genomes, clementine has 1,817 ECH-produced gene pairs (3,368 genes) from 86 blocks; sweet orange has 962 gene pairs (1,850 genes) from 54 blocks; pummelo has 1,402 gene pairs (2,604 genes) from 77 blocks, and citron and papeda has 39 paralogous blocks (471 gene pairs with 943 genes) and 43 paralogous blocks (443 gene pairs with 853 genes). The citrus relative, atalantia has 54 paralogous blocks with 790 gene pairs and 1,511 genes.

**Table 1:** Number of duplicated genes within a genome related to the ECH.

**Species**	***V. vinifera***	***C. sinensis***	***C. clementine***	***C. grandis***	***C. ichangensis***	***C. medica***	***A. buxifolia***
ECH-related	66	54	86	77	39	43	54
	1,289	962	1,817	1,402	471	443	790
	2,364	1,850	3,368	2,604	943	853	1,511

The reason why the paralogous genes of citron, papeda, and atalantia appear to retain fewer ECH-related collinear genes is likely the unfinished positioning of the genes to chromosomes and relatively poor assembly.

### Multiple Genome Alignment and Genomic Fractionation

To explore genome structural changes in the Rutaceae, using the above-distinguished orthology and outparalogy between genomes, we built multiple genome alignments represented with a table of orthologous and paralogous genes (Supplementary Table 5). In the table, 21 columns can be divided into three collinear gene groups (7 × 3 = 21), corresponding to tripled genes in the ECH. The first column is filled with grape gene IDs (24,283 ones) in their chromosomal order and chromosome by chromosome, and the following six columns are assigned to each of the six studied Rutaceae plants for each collinear gene group. In each column, a cell is filled with a collinear gene ID, or a dot meaning no inferred collinear correspondence. Within each group, the collinear genes in the compared genomes are orthologous, while the relationship of collinear genes between groups can be classified into two types, paralogous genes within the same species and outparalogous ones between different species. We showed the collinearity among these compared species in Supplementary Figure S2.

**Figure 2:**
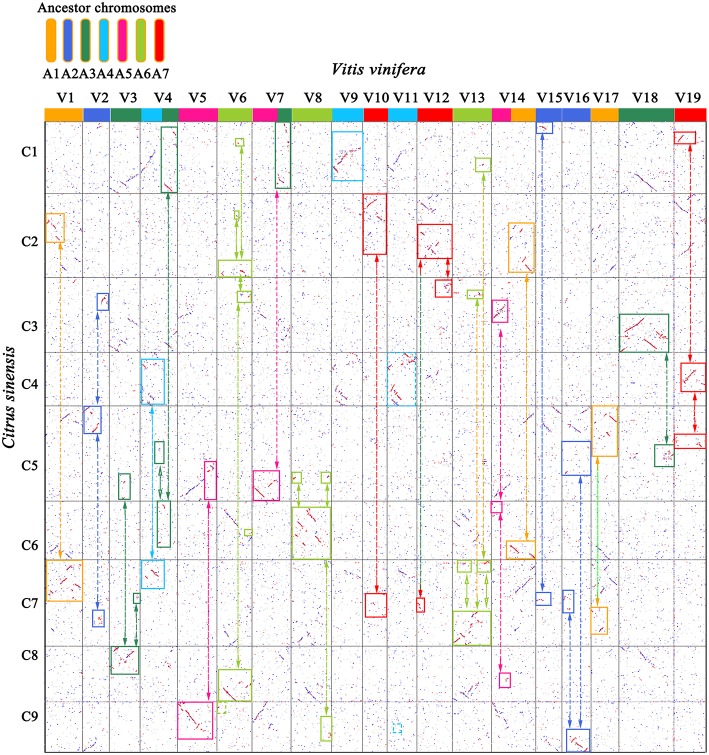
Genome homology between grape (V) and sweet orange (C). Best-matched genes from two genomes are colored in red, secondary matches in blue, and others in gray. The 19 grape chromosomes are shown in 7 colors, corresponding to their 7 ancestral proto-chromosomes before the ECH. Orthologous blocks were identified by solid-line rectangles. Bi-directional arrows link complementary correspondence showing inferred chromosome breakages during evolution of sweet orange.

Based on the homology table, we characterized genomic fractionation in each Rutaceae genome after its divergence from grape. With grape as the reference, we found 69 fragments (8,249 genes, 34.05% of the total) within sweet orange (Figure 2), 71 (9,670 genes, 39.91%) within clementine, and 71 (9,086 genes, 37.50%) within pummelo (Table 2). Using the constructed pseudo-chromosomes of three other Rutaceae plants, we inferred 72 fragments (5,341 genes, 22.04% of the total) within papeda, 77 (4,689 genes, 19.35%) within citron, and 82 (6,309 genes, 26.04%) within atalantia (Table 2). These findings show large-scale genomic fragmentation in Rutaceae plants (Supplementary Table 6). Additionally, we found that Rutaceae plants shared 87% (60 fragments) of collinear segments and breakage points with grape chromosomes, with sweet orange having the lowest (Figure 2), showing that most of the chromosomal rearrangement occurred before their divergence.

**Table 2:** Orthologous genes in citrus and their relative with grape as reference.

***V. vinifera*****(Vv)**		***C. sinensis*** **(Cs)**			***C. clementine*** **(Cc)**			***C. grandis*** **(Cg)**			***C. medica*** **(Cm)**			***C. ichangensis*** **(Ci)**			***A. buxifolia*** **(Ab)**		
**Chr**	**Genes**	**Frag. in Vv**	**Coli. genes**	**Perc. in Vv**	**Frag. in Vv**	**Coli. genes**	**Perc. in Vv**	**Frag. in Vv**	**Coli. genes**	**Perc. in Vv**	**Frag. in Vv**	**Coli. genes**	**Perc. in Vv**	**Frag. in Vv**	**Coli. genes**	**Perc. in Vv**	**Frag. in Vv**	**Coli. genes**	**Perc. in Vv**
Vv1	1,406	4	564	0.4	8	712	0.51	6	660	0.47	4	323	0.23	6	406	0.29	6	364	0.26
Vv2	976	3	397	0.41	3	382	0.39	2	347	0.36	2	172	0.18	2	289	0.3	3	329	0.34
Vv3	1,132	3	373	0.33	2	430	0.38	2	369	0.33	4	213	0.19	4	129	0.11	3	219	0.19
Vv4	1,333	7	560	0.42	4	518	0.39	4	479	0.36	6	270	0.2	9	436	0.33	9	329	0.25
Vv5	1,445	2	519	0.36	2	569	0.39	3	610	0.42	2	306	0.21	2	329	0.23	3	418	0.29
Vv6	1,289	7	554	0.43	6	631	0.49	4	584	0.45	8	284	0.22	8	390	0.3	8	411	0.32
Vv7	1,430	2	521	0.37	2	584	0.41	2	585	0.41	4	280	0.2	2	382	0.27	2	371	0.26
Vv8	1,488	6	540	0.36	7	755	0.51	6	674	0.45	6	315	0.21	5	394	0.26	7	382	0.26
Vv9	1,140	1	288	0.25	1	331	0.29	2	317	0.28	1	188	0.16	3	142	0.12	2	137	0.12
Vv10	894	3	292	0.33	3	339	0.38	3	292	0.33	3	91	0.1	3	144	0.16	2	269	0.3
Vv11	1,093	1	422	0.39	1	460	0.42	4	413	0.38	1	308	0.28	5	231	0.21	1	349	0.32
Vv12	1,299	3	353	0.27	3	430	0.33	4	418	0.32	2	149	0.11	3	206	0.16	4	249	0.19
Vv13	1,437	3	406	0.28	4	491	0.34	3	465	0.32	3	179	0.12	1	260	0.18	5	347	0.24
Vv14	1,625	6	610	0.38	8	660	0.41	7	636	0.39	5	398	0.24	6	397	0.24	5	525	0.32
Vv15	957	2	85	0.09	2	301	0.31	2	297	0.31	5	20	0.02	4	67	0.07	3	73	0.08
Vv16	1,076	5	283	0.26	5	358	0.33	3	296	0.27	5	157	0.15	4	153	0.14	5	220	0.2
Vv17	1,021	4	362	0.35	4	510	0.5	4	440	0.43	4	254	0.25	4	230	0.23	6	311	0.3
Vv18	2,007	2	757	0.38	2	792	0.39	3	770	0.38	2	582	0.29	2	554	0.28	2	705	0.35
Vv19	1,200	5	363	0.3	4	417	0.35	7	434	0.36	5	200	0.17	4	202	0.17	6	301	0.25
Total	24,248	69	8,249	0.34	71	9,670	0.4	71	9,086	0.37	72	4,689	0.2	77	5,341	0.22	82	6,309	0.26

*Chr, Chromosome; Frag., Fragments; Coli., Collinear; Perc., Percents*.

Gene collinearity due to gene loss and translocation before the radiation of the Rutaceae lineage was examined using sweet orange as the reference (Supplementary Table 7), with its 23,582 genes related to their orthologs and outparalogs from other Rutaceae plants, and paralogs in sweet orange itself. The lineage-specific gene content and genomic structural changes after the radiation of Rutaceae plants are illustrated in Supplemental Figure S3 with sweet orange as the outgroup. We counted the collinear genes with sweet orange as the reference–9 sweet orange chromosomes (23,582 genes) were mapped with 58 collinear fragments (13,848 genes, 58.72% of the total) in clementine; 85 (14,236 genes, 60.37% of the total) in pummelo; 49 (10,205 genes, 43.27% of the total) in citron; 68 (11,715 genes, 49.68%) in papeda; and 37 (12,643 genes, 53.61%) in atalantia (Table 3 and Supplementary Table 8). For example, sweet orange chromosome 1 was mapped with eight fragments in clementine; with two fragments each mapped to chromosomes 4 and 5, with three fragments each to chromosomes 1 and 3, with one fragment to chromosome 1 fragment being inverted.

**Table 3:** Orthologous genes in citrus and their relative with sweet orange as reference.

***C. sinensis*** **(Cs)**		***C. clementine*** **(Cc)**			***C. grandis*** **(Cg)**			***C. medica*** **(Cm)**			***C. ichangensis*** **(Ci)**			***A. buxifolia*** **(Ab)**		
**Chro**	**Chro_num**	**Frag. in Cs**	**Coli. genes**	**Perc. in Cs**	**Frag. in Cs**	**Coli. genes**	**Perc. in Cs**	**Frag. in Cs**	**Coli. genes**	**Perc. in Cs**	**Frag. in Cs**	**Coli. genes**	**Perc. in Cs**	**Frag. in Cs**	**Coli. genes**	**Perc. in Cs**
Cs1	2,690	8	1,546	0.57	10	1,551	0.58	3	1,073	0.4	8	1,187	0.44	6	1,333	0.5
Cs2	3,136	8	1,873	0.6	7	1,940	0.62	10	1,231	0.39	7	1,660	0.53	4	1,722	0.55
Cs3	2,799	5	1,550	0.55	10	1,616	0.58	6	1,284	0.46	6	1,360	0.49	5	1,459	0.52
Cs4	2,009	3	1,171	0.58	9	1,206	0.6	3	929	0.46	11	1,018	0.51	1	1,187	0.59
Cs5	3,554	6	2,091	0.59	16	2,235	0.63	8	1,559	0.44	8	1,708	0.48	5	1,899	0.53
Cs6	2,200	5	1,371	0.62	5	1,328	0.6	1	1,116	0.51	6	1,268	0.58	4	1,169	0.53
Cs7	3,229	13	1,992	0.62	19	2,017	0.62	6	1,327	0.41	9	1,736	0.54	5	1,883	0.58
Cs8	2,067	5	1,163	0.56	6	1,232	0.6	2	859	0.42	8	920	0.44	4	931	0.45
Cs9	1,898	5	1,091	0.57	3	1,111	0.59	10	827	0.44	5	858	0.45	3	1,060	0.56
Total	23,582	58	13,848	0.59	85	14,236	0.6	49	10,205	0.43	68	11,715	0.5	37	12,642	0.54

*Chr, Chromosome; Frag., Fragments; Coli., Collinear; Perc., Percents*.

### Gene Loss and Retention

To better explain the collinearity in citrus genomes, we selected a partial segment of multiple gnome alignments to exhibit gene content changes (Supplementary Figure S4). Aligned homologous regions were selected from grape chromosomes 14, 1, and 17, best-matched/orthologous to local regions of Rutaceae chromosomes 6, 7 and 5, respectively. A region of 0.75 Mb on grape chromosome 14 (from 29.55 to 30.25 Mb) shares an appreciable number of orthologous genes (70 genes) with sweet orange chromosome 6 (31 genes, 20.52–20.96 Mb), the orthologous region in clementine chromosome 6 (31 genes, 24.92–25.39 Mb) and the orthologous region in pummelo chromosome 6 (29 genes, 23.07–23.50 Mb). Unlike the former ones, the above region on grape chromosome 14 shares a relatively short fragment on citron chromosome 6 (17 genes, 37.53–37.82 Mb), a dispersed orthology in atalantia (38 genes, 49.52–50.76 Mb) and papeda (30 genes, 42.12–42.60 Mb).

The first group taking grape chromosome 14 as the reference showed a higher collinear gene density than the other two groups of outparalogs that were more dispersed. In the second group of local alignments, the long region on grape chromosomes 1 (11.01–12.39 Mb) aligned with chromosome 7 in citrus and their relative, specifically located on sweet orange (12 genes, 1.62–2.11 Mb), clementine (11 genes, 23.62-24.03 Mb), pummelo (11 genes, 20.40-20.80 Mb), citron (8 genes, 5.31–5.64 Mb), papeda (9 genes, 2.51–3.35 Mb), and atalantia (8 genes, 23.41–23.78 Mb).

The last group of homologous regions produced by ECH showed a relatively regular alignment, with the region on grape chromosome 17 (6.17–6.61 Mb) corresponding to the regions on chromosome 5 (7 collinear genes located) in Rutaceae plants in a region about 2–6 Mb (7–9 genes) apparently located at the end of the chromosome. More detailed information about the local alignment among the compared species is displayed in Supplementary Figure S4.

Genomic fragmentation was accompanied by wide-spread gene losses or translocations. Using the grape genome as a reference, 37.5%-80.6% of genes were retained as collinear orthologs. For example, using grape chromosome 1 as outgroup, 65.2, 84.7, and 78.4% grape genes were found to have collinear counterparts in sweet orange, clementine, and pummelo, respectively (Supplementary Table 9). The other three Rutaceae plants have lower gene retention rates likely due to poor assembly. To better display the scale of gene retention, we depicted the retained genes within Rutaceae plants after the divergence with grape (Supplementary Figures S5, S6.)

**Figure 3:**
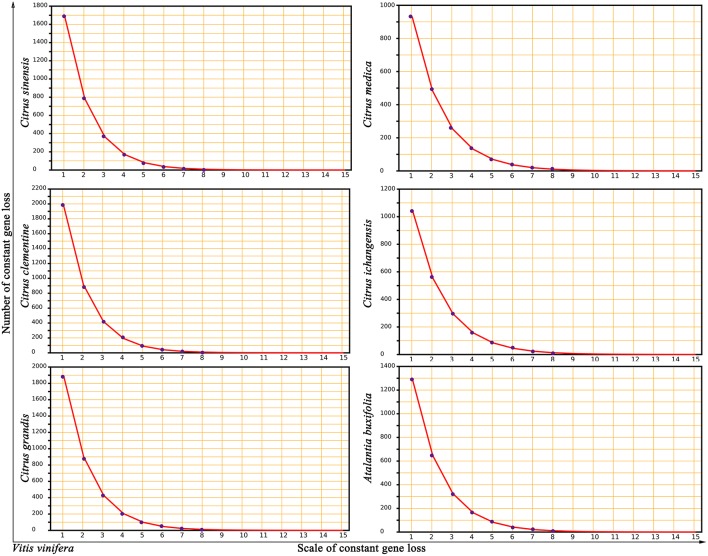
Near geometric distribution of continually lost or translocated genes between grape and Rutaceae plants. *Vitis vinifera* was compared to 5 citrus (*Citrus sinensis, Citrus clementine, Citrus grandis, Citrus medica*, and *Citrus ichangensis*) and one relative, *Atalantia buxifolia*. The x-axis represents numbers of continually lost or translocated genes in the inferred homologous regions. The y-axis represents the number of removed genes.

To investigate potential mechanisms of genomic fractionation, we counted the number of removed genes (due to gene deletion or translocation) in each Rutaceae genome. We inferred that 3,765 genes were removed from the orthologous regions in sweet orange; and similar numbers were detected in other plants (4,221 in clementine, 4,143 in pummelo, 2,580 in citron, 2,788 in papeda, and 3,139 in atalantia). By checking the gap sizes between neighboring genes in collinearity, we found that most (87–96%) gene removal events involved 15 or fewer genes, with the majority of removals involving one or two genes in an event. A statistical fitness regression showed that gene removal patterns largely followed a geometric distribution (geometric parameter 0.4676–0.5396 and goodness of fit *F*-test *p*-value 0.8852–0.9356 to accept the fitness) (Figure 3). For example, we inferred that 10.22% (2,409) DNA fractionation events are small in scale, removing 1 or 2 genes at one time, and responsible for 63.98% of the removed (3,765) genes in sweet orange.

We also characterized gene loss and retention in Rutaceae plants with sweet orange as the reference (Supplementary Figures S7, S8). Compared to sweet orange, 76.0–82.6% orthologous genes were retained in clementine, and similar numbers in pummelo (78.6–84.2%). Much lower inferred retention rates in citron (52.2–67.6%), papeda (60.6–76.6%), and atalantia (61.6–79.6%) may be the result of insufficient genome assemblies (Supplementary Table 10). The number of continually removed genes in the other Rutaceae plants also approximately followed a geometric distribution, with parameters from 0.5991 to 0.6661, fitness values from 0.9908 to 0.9986, and *p*-values (*F*-test) from 0.9093 to 0.9635 (Supplementary Table 11). Events removing 1 or 2 genes accounted for most removal events [clmentine (77.30%), pummelo (79.82%), citron (70.16%), papeda (65.55%), and atalantia (74.43%)].

### Evolutionary Divergence and Dating

**Figure 4:**
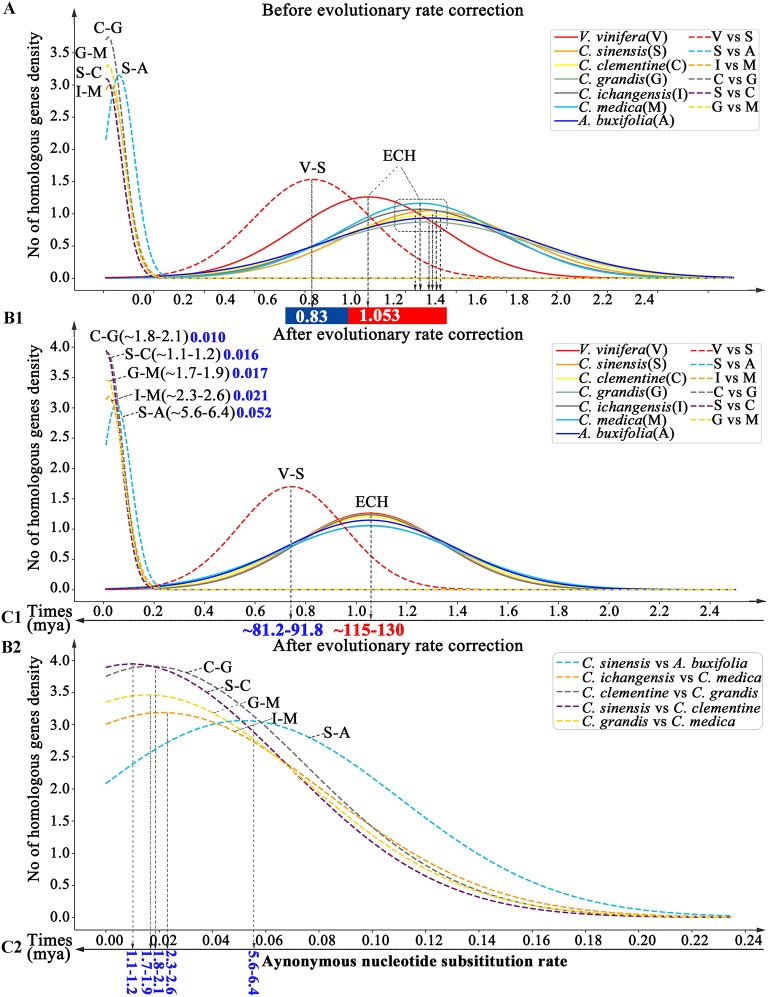
Dating evolutionary events within and among Rutaceae genomes. Distributions of Ks between intragenomic collinear genes are shown with solid curves and intergenomic ones with dashed curves. **(A)** Distributions before Ks correction. **(B1,2)** and **(C1,2)**. Distributions of Ks after correction. Inferred times are shown beneath Time-axis.

By inferring Ks between paralogous and orthologous genes in collinearity, we estimated divergence time among Rutaceae plants. We found that the ECH Ks distributions in different Rutaceae plants had peak locations ranging from 1.252–1.301, with papeda at Ks = 1.252 (±0.343), and sweet orange at Ks = 1.301 (±0.343). This shows a <5% difference in evolutionary rates among Rutaceae plants. By contrast, Rutaceae plants evolved faster (18.90–23.55%) than grape with Ks peak of its paralogs at 1.053 (±0.29) (Supplementary Table 12 and Figure 4). Using grape as the reference, we aligned the Rutaceae Ks peaks to its peak and performed an evolutionary rate correction (See Methods for details). Then, using the corrected Ks, we assessed evolutionary dates of speciation events. The Ks peak of grape-Rutaceae orthologs was 0.744(±0.21). Assuming that the ECH occurred ~115–130 mya, the split of Rutaceae from grape and other eudicots was inferred to be 81.15–91.74 mya (Supplementary Table 13 and Figure 4). Rutaceae plants radiated <10 mya. For example, *Atalantia buxifolia*, belonging to the genus related to citrus, the divergent node with citrus appeared at 5.6–6.4 mya, while the remaining species split within the last 2 million years. Especially recent was the divergence time between sweet orange and clementine (1.1–1.2 mya), and pummelo and clementine (1.8–2.1 mya).

### Vitamin C Synthesis

We investigated how the ECH affected the copy number variation of genes involved in the biosynthesis of vitamin C. Using previously reported genes related to the biosynthesis of sweet orange vitamin C as seeds (Xu et al., 2013), we found their homologous genes in Rutaceae genomes with Blastp E-value <1e-5, score >150 and identity ≥50 (Altschul et al., 1990) (Supplementary Table 14). We identified total numbers of vitamin C gene homologs in Rutaceae plants ranging from 101 (sweet orange) to 143 (papeda). In grape, 53.39% (63) of vitamin gene homologs are ECH-related, showing that polyploidization increased vitamin C gene copy numbers. In citron (26 genes, 23.01%) and papeda (31, 24.41%), relatively smaller percentages of vitamin C genes are ECH-related, possibly underestimated due to incomplete genome assembly. For example, L-ascorbate peroxidase (APX) involves 4 ECH-related copies in every Rutaceae plant. The biggest subfamily, Putative pectinesterase/pectinesterase inhibitor (PME), has 35 genes in clementine, and 10 copies are ECH-related (~29%). The subfamilies, L-galactono-1,4-lactone dehydrogenase (GLOase) and L-galactono-1,4-lactone dehydrogenase (GalLDH) have no genes clearly related to ECH in Rutaceae plants, while in grape, they have ECH-related copies (Supplementary Table 14).

**Figure 5:**
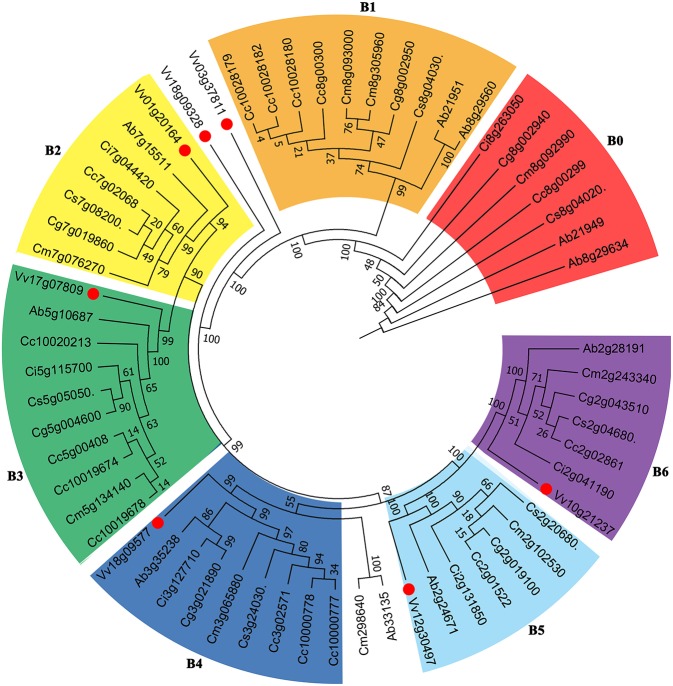
Gene tree of Vitamin C related genes. The nucleobase-ascorbate transporter (AAT) gene family including 61 homologous copies in grape and Rutaceae plants was constructed. Numbers displayed in the nodes represent the percent bootstrap from 1,000 repetitions.

We constructed phylogenetic gene trees to help understand the evolution of genes involved in the biosynthesis of vitamin C. The trees displayed complicated relationships just like their ambiguous hybrid relationships among these compared Rutaceae plants (Supplementary Figures S9, S10). Here we show a gene tree of the subfamily within the Vitamin C pathway, nucleobase-ascorbate transporter (AAT) (Figure 5). The genes can be divided into several groups, and we selected 6 larger groups marked B1-B6. From B6, taking grape genes as an outgroup, we observed that the relationship of genes Vv10g21237- Ab2g28191- Ci2g041190- Cm2g243340- Cg2g043510- Cs2g0680- Cc2g02861 basically agrees with the phylogenetic tree of plants. We have to note that subtrees formed by Rutaceae genes displayed complex relationships among these plants, especially between citron and “the triangle” among sweet orange (Cs), clementine mandarin (Cc), and pummelo (Cg). Since the genetic hybridization within “the triangle,” the three citrus plants tend to run faster than the wild citrus, citron (Cm), which made certain subtrees inconsistent with their evolutionary relationship (Figure 5). More specific information is displayed in Supplementary Figures S9, S10.

## Discussion

### Genome Fractionation

Polyploidization, at the whole genome level, increased gene content, subsequently even immediately followed by enormous genomic changes, e.g., chromosome fragmentation and/or rearrangement, potential gene deletion or relocation, gene mutation and origination of novel genes (Soltis and Soltis, 1999; Blomme et al., 2006; Soltis et al., 2009; Sankoff and Zheng, 2012). Comparing six Rutaceae plants and grape (preserving a relatively complete genome comparing with the ECH), we found frequent fractionations and rearrangements. That is, these Rutaceae genomes suffered appreciable changes after the divergence with grape. Unlike other extant plants that have experienced multiple rounds of polyploidization, Rutaceae have instead hybridized with their relatives since the ECH over 100 million years ago. The hybridization may also contribute to genome fractionation due to homologous recombination.

### High Gene Retention Rate in Rutaceae

Large scale gene losses have been ascribed to polyploidization (Bowers et al., 2003; Town et al., 2006; Schnable et al., 2012), and previous studies found a gene loss rate of 70% within the cotton genome after decaploidy, showing genomic instability (Wang et al., 2016). Redundant copies of genes after polyploidization are often removed from the genome by fractionation (Langham et al., 2004; Schnable et al., 2012). However, with obvious deviation from a random distribution, gene losses could be more complex. Rutaceae plants may have suffered by recursive gene losses accompanied by fractionation, and the ongoing manner of gene loss appeared in extant species was reported (Scannell et al., 2006; Swanson-Wagner et al., 2010; Woodhouse et al., 2010). In this study, we found that the better assembled Rutaceae genomes had collinear gene retention rates up to 65% or more. Comparatively, plants with extra rounds of polyploidization tended to have less gene retention, e.g., upon to 31% in soybean, which was affected by two additional tetraploidization events after the ECH (Wang et al., 2017a), and 58.4% in watermelon, affected by one additional tetraploidization (Wang et al., 2018).

### Divergent Evolutionary Rates

Considering the controversial origins of Rutaceae plants, we selected duplicated genes produced by polyploidization within a genome, or collinear orthologous genes between two different species, to deduce their evolutionary rates. Plants often evolve at very divergent rates. A previous study in cucurbits, using the above mentioned approach, proved that melon is the slowest at evolving, with watermelon and cucumber faster by 23.6 and 27.4% (Wang et al., 2018). Likewise with legumes, lotus evolves the slowest and peanut was nearly 25% faster (Wang et al., 2017a). The studied homologous blocks and the collinear genes residing in them are almost sure to have originated simultaneously, providing a good opportunity to evaluate divergent evolutionary rates among genomes. Here, with ECH-related duplicated genes in Rutaceae plants, we found Rutaceae plants varying by <5% in their evolutionary rates, and showing a 19–24% faster rate than grape. Divergent evolutionary rates may be related to their ecological niche, and their different evolutionary history. The actual biological mechanisms responsible for divergent evolutionary rates remain to be identified.

## Data Availability

The raw data supporting the conclusions of this manuscript will be made available by the authors, without undue reservation, to any qualified researcher.

## Author Contributions

XW and JW: conceptualization. JYuan and JW: formal analysis. JYu, FM, YZ, JL, PS, SSu, ZZ, CL, CW, HG, XL, XD, SSh, YX, YH, JZ, and TS: formal analysis. JYuan: data curation. JYuan and JW: writing—original draft. XW: writing—review and editing. XW: supervision.

### Conflict of Interest Statement

The authors declare that the research was conducted in the absence of any commercial or financial relationships that could be construed as a potential conflict of interest.
