# Supplementary material for: Alignment of Rutaceae Genomes Reveals Lower Genome Fractionation Level Than Eudicot Genomes Affected by Extra Polyploidization
Source: Front Plant Sci. 2019 Aug 6;10:986. doi: 10.3389/fpls.2019.00986 (PMC6691040; doi:10.3389/fpls.2019.00986)
Supplement: Supplementary file 1 [file Data_Sheet_1.docx]

***Supplementary Material***

# Supplementary Data

Supplementary Material includes Supplementary Figures S1-S8, Supplementary Tables S1, S3-4, S9-14. The left Supporting Tables (S2, S5-8) showed in supplemental excel tables.

# Supplementary Figures and Tables

## **Supplementary Figures**


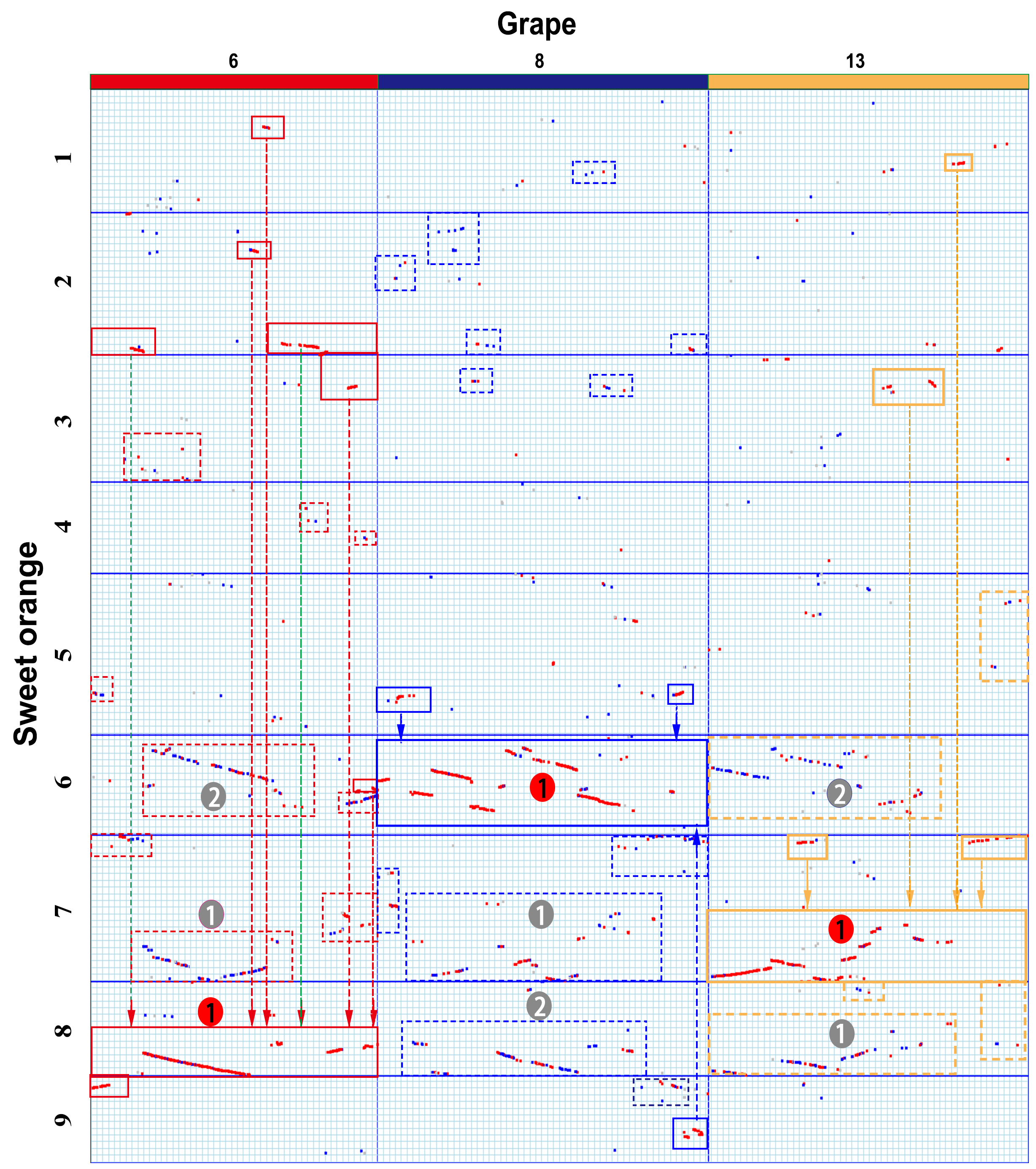


**Supplemental Figure S1. Dot plots between grape and sweet orange.** Grape chromosome 6, 8 and 13 are homo(eo)logous chromosomes produced by hexaploidy common to the major eudicot plants. Dots produced by best matched genes from two genomes are colored in red, secondary matched dots in blue, and the other in grey. Orthologous blocks between grape and sweet orange are enclosed by solid-line rectangles marked by “1” in red solid-circles, and outparalogous blocks by dashed rectangles marked by “1” or “2” in grey solid-cricles. The sweet orange regions orthologous to one grape chromosome are outparalogous to the other two grape chromosomes.


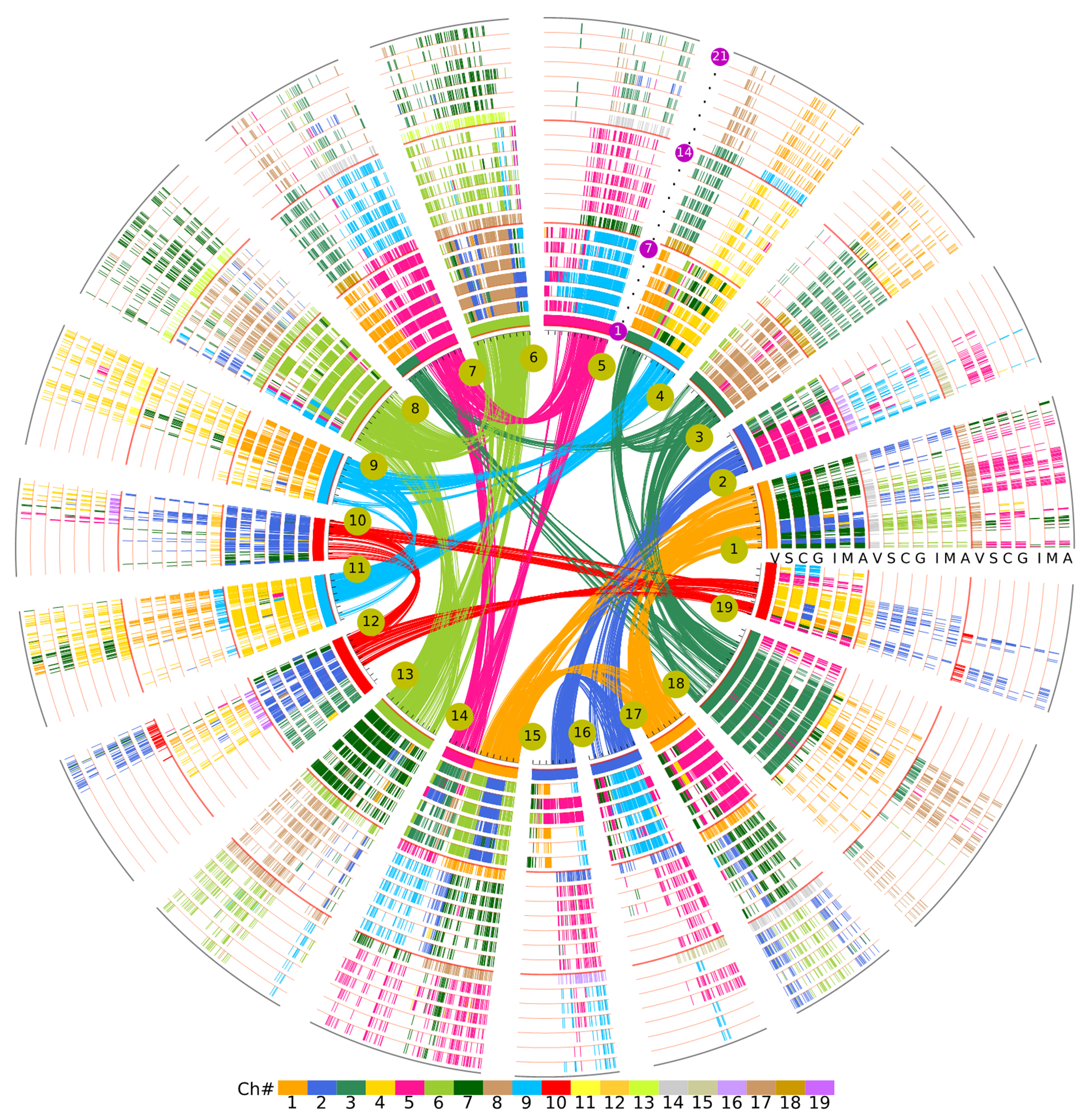


**Supplemental Figure S2. Pan-genome homology alignments of Rutaceae plants with grape as reference.** Grape chromosomes are displayed in the inner circle shown in 7 colors, corresponding to their 7 ancestral proto-chromosomes before the hexaploidization. A total 21 circles are separated into three groups, in which the first inner group represent the orthologous correspondence between genomes, and the two outer groups corresponding to the outparalogs.


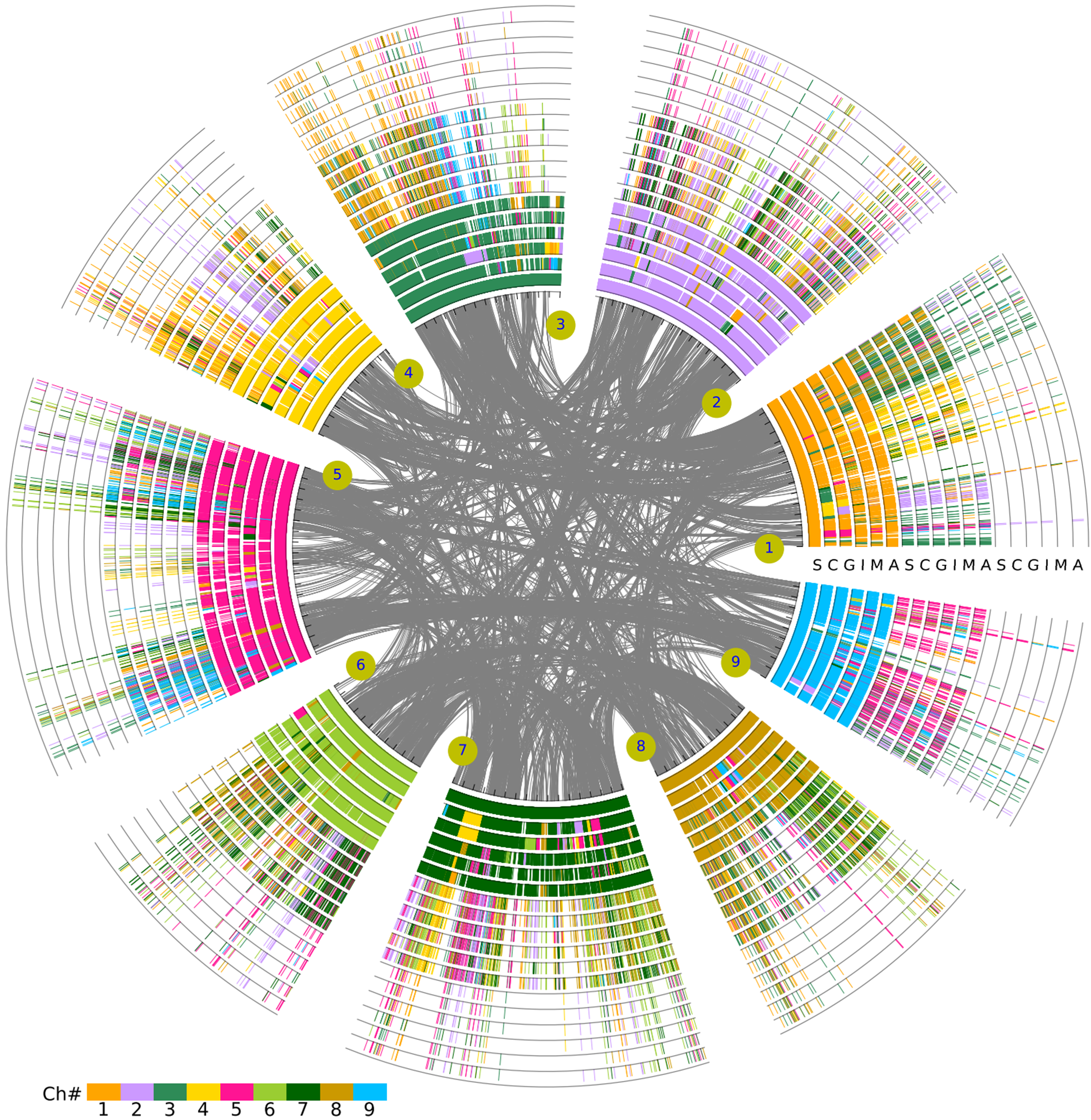


**Supplemental Figure S3.** **Homologous alignments of within citrus and citrus relative.** Sweet orange (S) as outgroup genome was used to compare with 4 citrus and one relative to exhibit their collinearity. Sweet orange displayed in the inner circle with 9 chromosomes divided into three groups caused by ECH colored by the same color. All the 18 circles were separated into three groups, in which the first inner group represent the orthologs between each other and the two outer groups are outparalogs corresponding to the first group respectively. The short colored bars in the circle represent the homologous genes between the compared genomes. The inner Bezier curves connected the genes distributed in sweet orange genomes showing that these genes were produced by ECH.





**Supplemental Figure S4.** **Alignment of local regions sharing homology.** *Vitis vinifera* (Vv), *Citrus sinensis* (Cs), *Citrus clementine* (Cc), *Citrus grandis* (Cg), *Citrus ichangensis* (Ci), *Citrus medica* (Cm) And *Atalantia buxifolia* (Ab) homologous genes located in the local collinear regions shows their core eudicot-common ancestor undergone polyploidization. The seven species of each group (A, B, and C) represents orthologs within that group. The same species in groups B and C are paralogs to group A, while different species in groups B and C are outparalogs to group A.


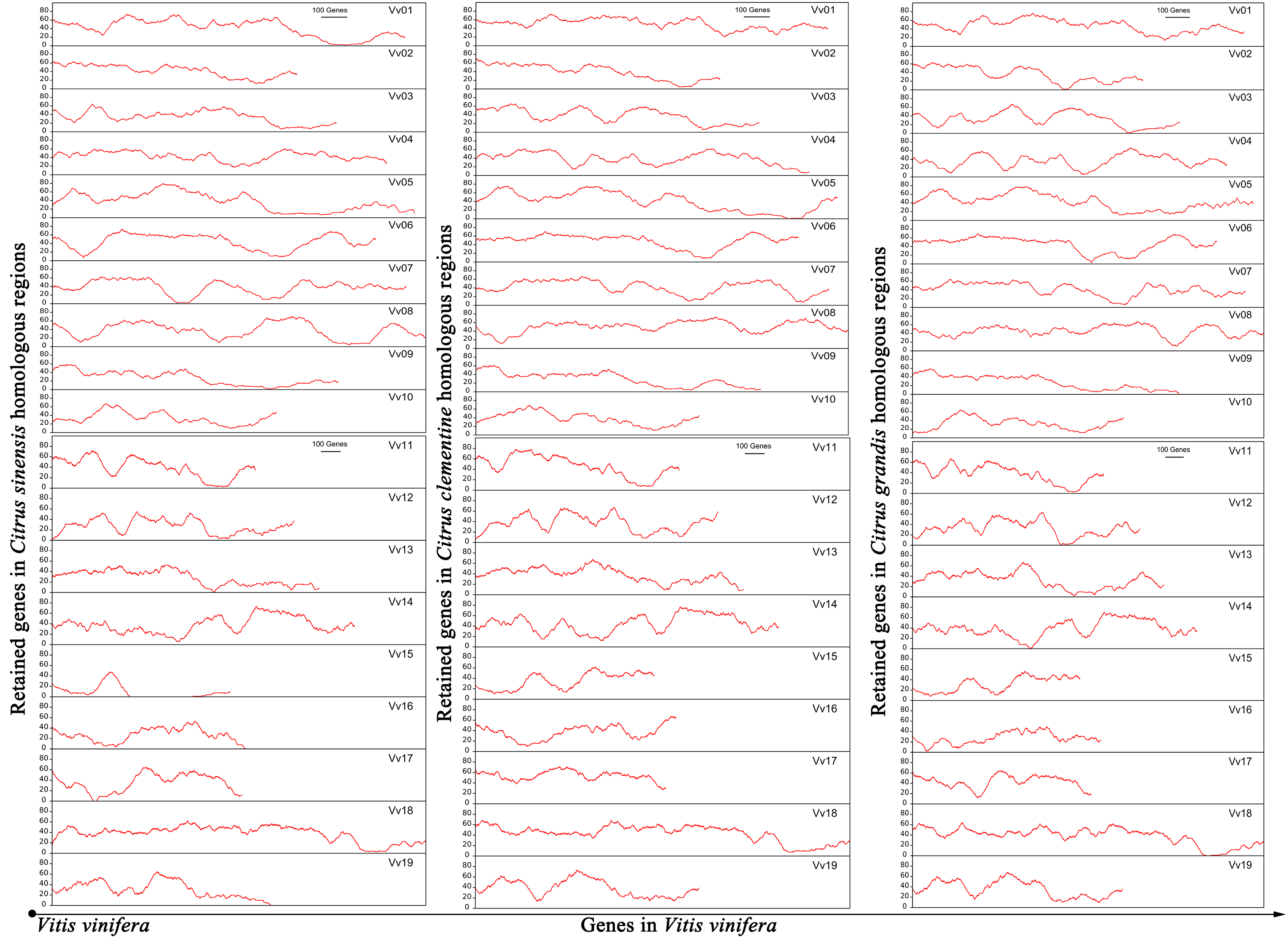


**Supplementary Figure 5. Retention of *Citrus sinensis*, *Citrus clementine* and *Citrus grandis* along with their orthlogous *Vitis vinifera* chromosome.** Rates of retained genes in sliding windows of citrus homologous regions.


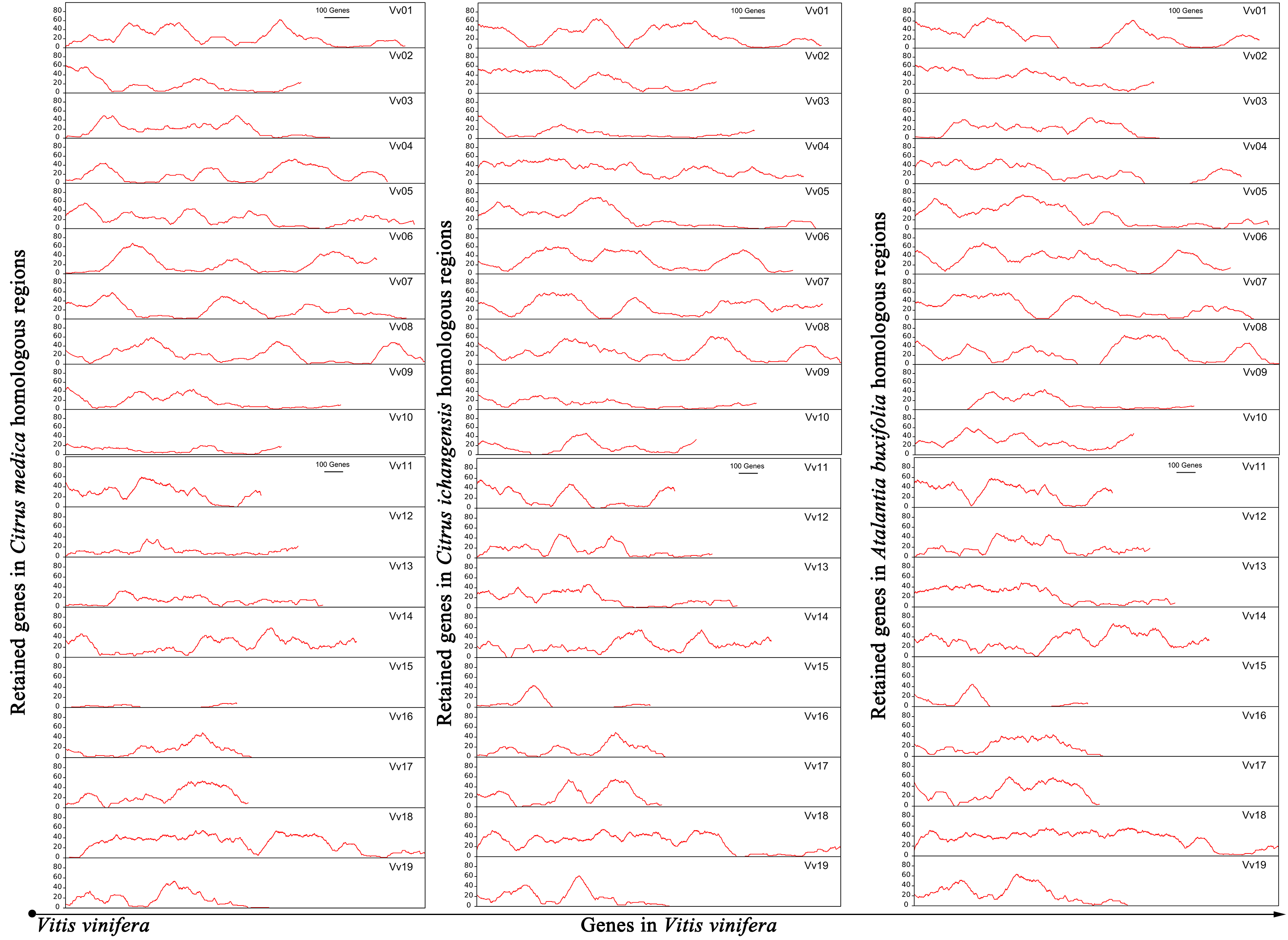


**Supplemental Figure S6.** **Retention of *Citrus medica*, *Citrus ichangensis* and *Atalantia buxifolia* along with their orthlogous *Vitis vinifera* chromosome.** Rates of retained genes in sliding windows of 2 citrus and one relative homologous regions.


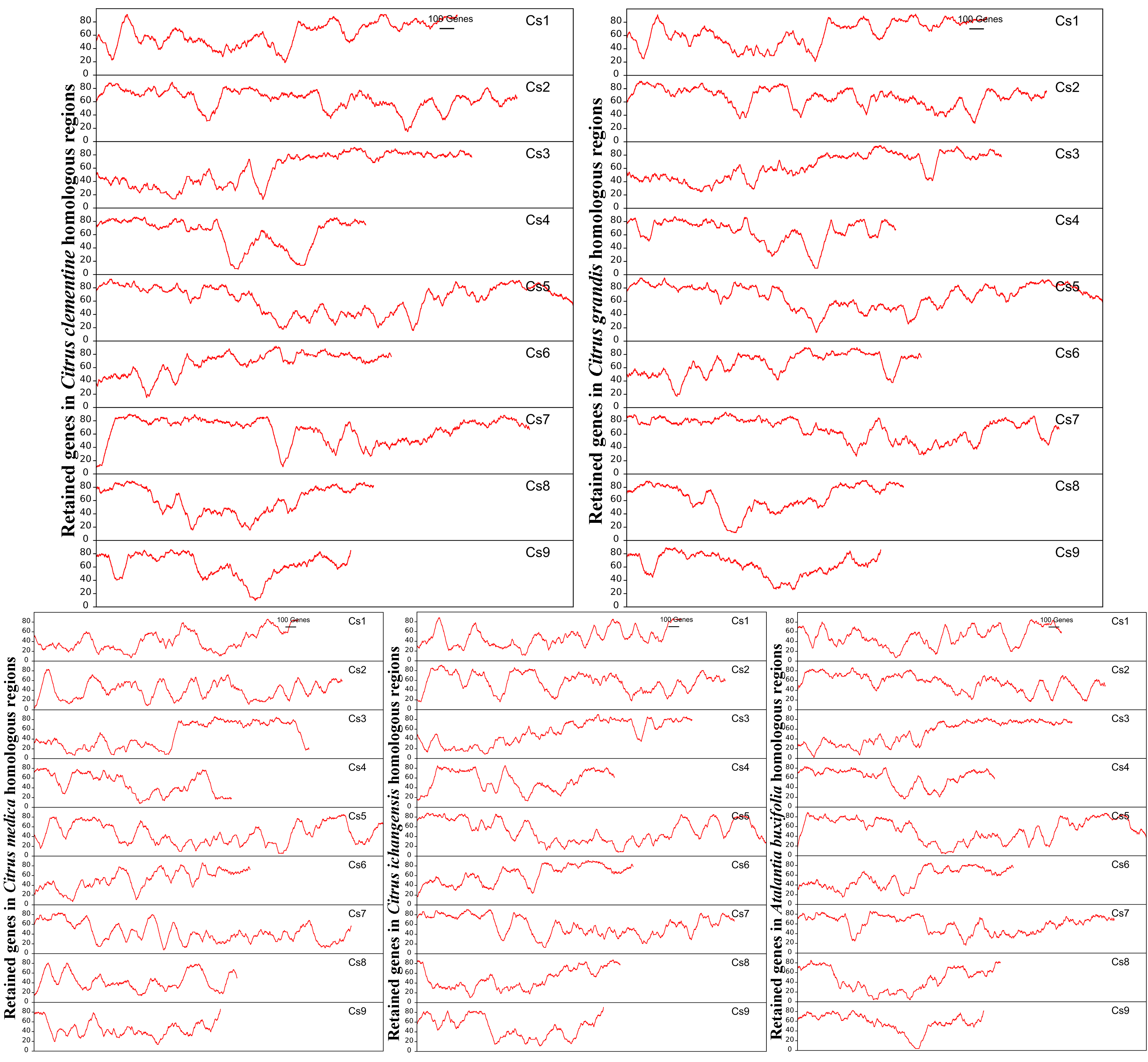


**Supplemental Figure S7.** **Retention of *Citrus clementine*, *Citrus grandis*, *Citrus medica*, *Citrus ichangensis* and *Atalantia buxifolia* along with their orthologous**

***Citrus sinensis* chromosomes.** Rates of retained genes in sliding windows of 4 citrus and one relative homologous regions.


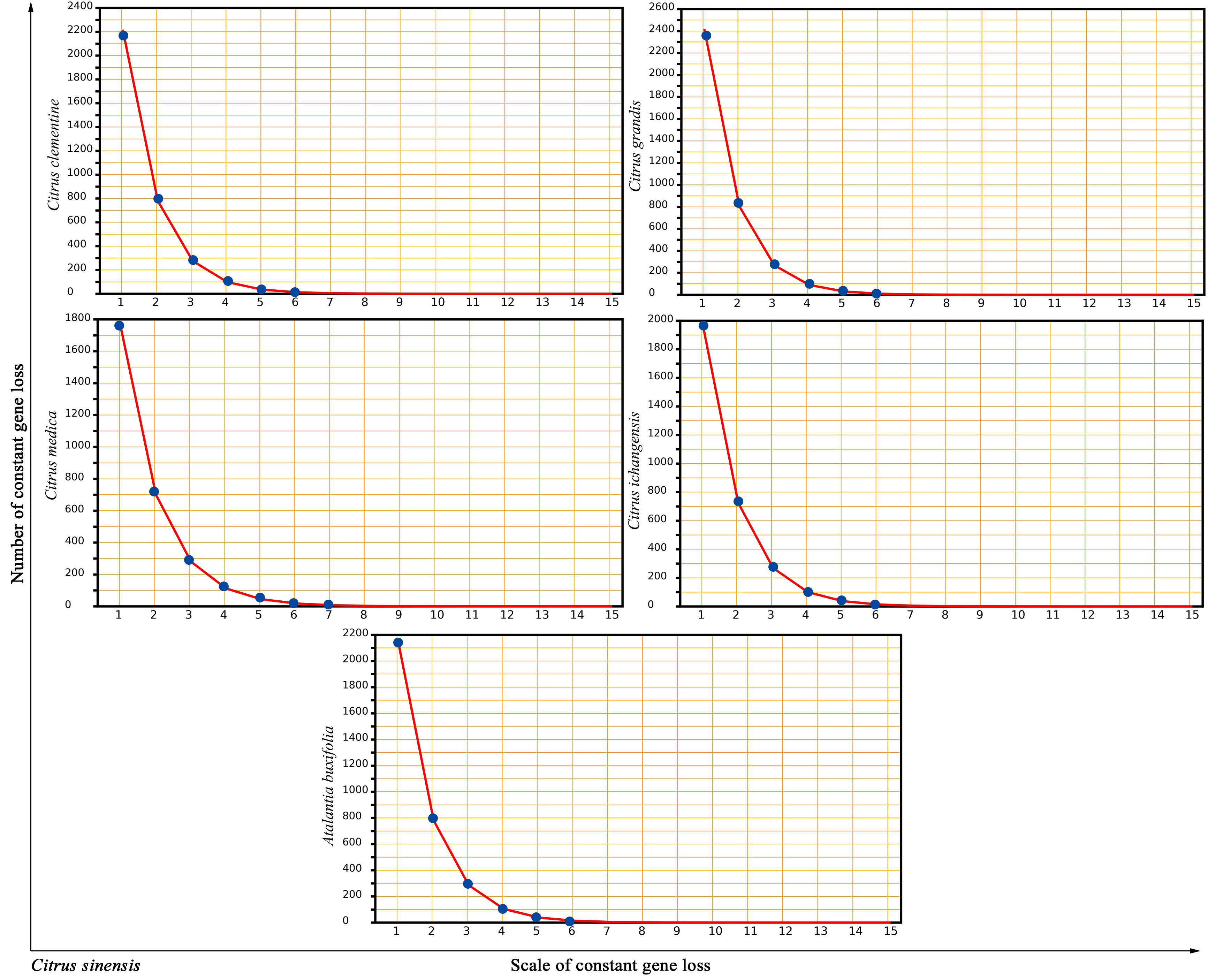


**Supplemental Figure S8.** **Fitting a geometric distribution in gene loss within citrus and relative.** The figure systematically compared Citrus sinensis to 4 citrus (*Citrus clementine*, *Citrus grandis*, *Citrus medica* and *Citrus ichangensis*) and one relative, *Atalantia buxifolia*. The x-axis represents numbers of connected gene deletion in the collinear regions, where we mainly selected constant one deleted gene to 15 deleted genes. The y-axis represents the account number of each scale of deleted genes.


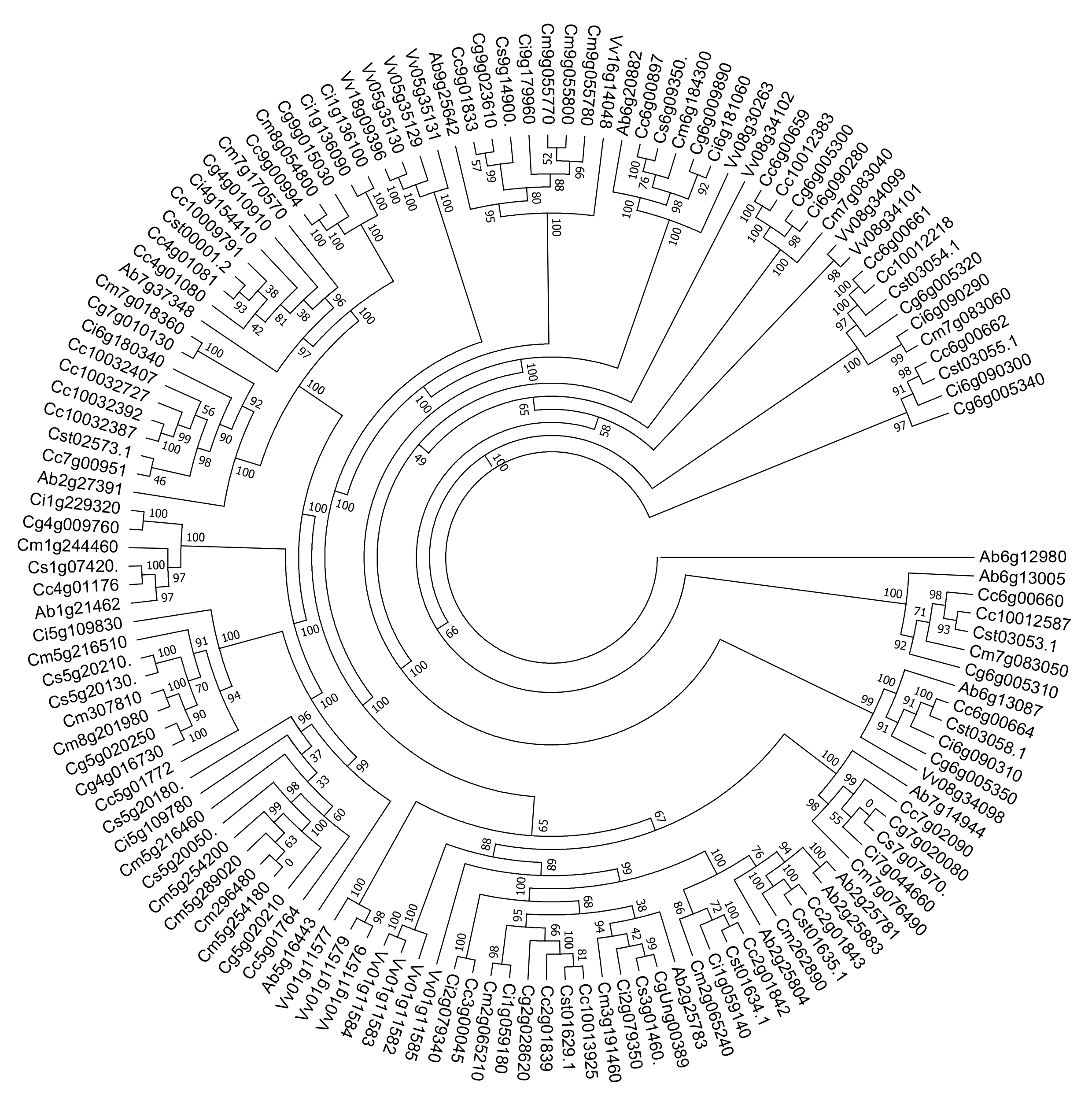


**Supplemental Figure S9. Gene tree of Vitamin C related genes.** The family of d-galacturonic acid reductase (GalUR) including 136 homologous copies in grape and Rutaceae plants was constructed. Numbers displayed in the nodes represent the percent bootstrap from 1000 repetitions.


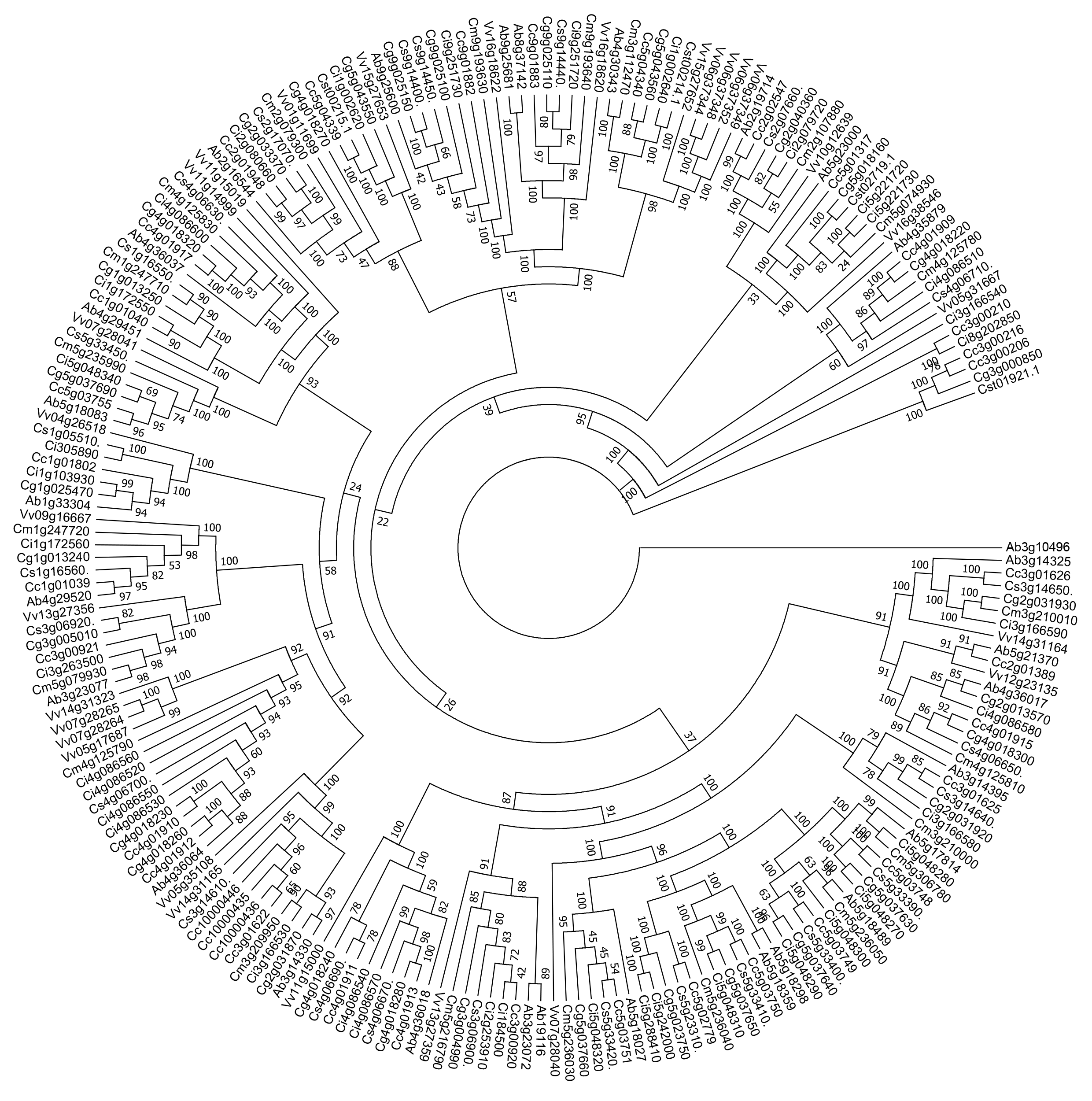


**Supplemental Figure S10**. **Gene tree of Vitamin C related genes.** The family of PME including 214 homologous copies in grape and Rutaceae plants was constructed. Numbers displayed in the nodes represent the percent bootstrap from 1000 repetitions.

## **Supplementary Tables**

**Supplementary Table 1. Information of genome data.**

| **Species name** | **Common name** | **Version** | **Genes** | **Genes on chr** | **Data source** | **Reference** |
| --- | --- | --- | --- | --- | --- | --- |
| ***Vitis vinifera*** | Grape | V3 | 26,346 | 24,283 | JGI  (https://phytozome.jgi.doe.gov/pz/portal.html) | Jaillon, O, Aury, J. M, Noel, B, Policriti, A, Clepet, C, Casagrande, A, et al, 2007(Jaillon et al., 2007) |
| ***Citrus sinensis*** | Sweet orange | V1 | 29,655 | 23,582 | CSAP  (http://citrus.hzau.edu.cn/orange/index.php) | Qiang Xu#, Ling-Ling Chen#, Xiaoan Ruan#, Di-Jun Chen, Andan Zhu, et al, 2013(Xu et al., 2013) |
| ***Citrus clementina*** | Clementina | V1.0 | 24,533 | 24,357 | JGI  (https://phytozome.jgi.doe.gov/pz/portal.html) | Middleton C P, Senerchia N, Stein N, et al, 2014(Wu et al., 2014) |
| ***Citrus grandis*** | Pummelo | V1 | 30,123 | 28,043 | CSAP  (http://citrus.hzau.edu.cn/orange/index.php) | Xia Wang#, Yuantao Xu#, Siqi Zhang#, Li Cao# et al, 2017(Wang et al., 2017) |
| ***Citrus ichangensis*** | Ichang papeda | V1 | 32,578 | 28,047 | CSAP  (http://citrus.hzau.edu.cn/orange/index.php) | Xia Wang#, Yuantao Xu#, Siqi Zhang#, Li Cao# et al, 2017(Wang et al., 2017) |
| ***Citrus medica*** | Citron | V1 | 32,067 | 27,017 | CSAP  (http://citrus.hzau.edu.cn/orange/index.php) | Xia Wang#, Yuantao Xu#, Siqi Zhang#, Li Cao# et al, 2017(Wang et al., 2017) |
| ***Atalantia buxifolia*** | Atalantia | V1 | 28,420 | 25,478 | CSAP  (http://citrus.hzau.edu.cn/orange/index.php) | Xia Wang#, Yuantao Xu#, Siqi Zhang#, Li Cao# et al, 2017(Wang et al., 2017) |

**Supplementary Table 3. Number of homologous blocks and gene pairs within a genome or between genomes.**

| Homologous Blocks within  and among genome | BL> 4 | BL > 10 | BL > 20 | BL > 50 | ACGP | LDB | LDB on chromosomes |
| --- | --- | --- | --- | --- | --- | --- | --- |
| *Vitis vinifera* | 2,116/176 | 1,425/61 | 999/33 | -/- | 12.02, 23.36,30.27,- | 46 | Vv1-Vv14/Vv4-Vv18 |
| *Citrus sinensis* | 2,144/227 | 1,115/55 | 635/21 | 129/2 | 9.44, 20.27, 30.24, 64.50 | 67 | Cs1-Cs3 |
| *Citrus clementine* | 2,856/243 | 1,849/69 | 1,386/37 | 460/7 | 11.75, 26.80, 37.46, 65.71 | 91 | Cc5-Cc9 |
| *Citrus grandis* | 2,728/288 | 1,360/57 | 890/25 | 228/3 | 9.47, 23.86，35.60, 76.00 | 106 | Cg5-Cg9 |
| *Citrus ichangensis* | 2,135/304 | 478/33 | 115/5 | -/- | 7.02, 14.48, 23.00, - | 26 | Ci5-Ci7 |
| *Citrus medica* | 2,110/307 | 376/24 | 63/2 | -/- | 6.87, 15.67, 31.50, - | 36 | Cm5-Cm9 |
| *Atalantia buxifolia* | 2,443/287 | 1,059/59 | 415/13 | 115/2 | 8.51, 17.95, 31.92, 57.50 | 61 | Ab5-Ab7 |
| *V. vinifera vs C. sinensis* | 15,891/1,096 | 11,257/307 | 9,099/161 | 6,141/64 | 14.50, 36.67, 56.52, 95.95 | 306 | Vv18-Cs3 |
| *V. vinifera vs C. clementine* | 17,661/1,070 | 13,172/294 | 11,382/176 | 8,183/75 | 16.51, 44.80, 64.67, 109.11 | 317 | Vv18-Cc3 |
| *V. vinifera vs C. grandis* | 16,394/1,018 | 12,218/307 | 10,375/177 | 6,623/63 | 16.10, 39.80, 58.05, 105.13 | 287 | Vv18-Cg3 |
| *V. vinifera vs C. ichangensis* | 16,080/1,344 | 10,245/390 | 7,043/169 | 2,851/36 | 11.96, 26.27, 41.67, 79.19 | 209 | Vv18-Ci3 |
| *V. vinifera vs C. medica* | 15,273/1,342 | 9,343/376 | 6,114/153 | 2,424/32 | 11.38, 24.85, 39.96, 75.75 | 210 | Vv18-Cm3 |
| *V. vinifera vs A. buxifolia* | 16,700/1,189 | 11,725/350 | 9,310/185 | 5,288/54 | 14.05, 33.31, 50.32, 97.93 | 239 | Vv07-Ab5 |
| *C. sinensis vs C. clementine* | 21,929/1,111 | 16,951/238 | 15,689/150 | 12,650/50 | 19.74, 71.22, 104.59, 253.00 | 1,328 | Cs3-Cc3 |
| *C. sinensis vs C. grandis* | 21,999/1,139 | 16,963/270 | 15,476/169 | 12,029/60 | 19.31, 62.83, 91.57, 200.48 | 864 | Cs5-Cg5 |
| *C. sinensis vs C. ichangensis* | 19,440/1,311 | 13,387/286 | 11,603/163 | 9,063/79 | 14.83, 46.81, 71.18, 114.72 | 437 | Cs1-Ci1 |
| *C. sinensis vs C. medica* | 17,750/1,321 | 11,331/229 | 9,619/109 | 7,919/54 | 13.44, 49.48, 88.25, 146.65 | 388 | Cs7-Cm7 |
| *C. sinensis vs A. buxifolia* | 21,116/1,301 | 15,097/259 | 13,545/152 | 11,138/74 | 16.23, 58.29, 89.11, 150.51 | 924 | Cs5-Ab5 |
| *C. clementine vs C. grandis* | 23,911/1,118 | 18,850/233 | 17,684/154 | 14,549/55 | 21.39, 80.90, 114.83, 264.53 | 1,356 | Cc5-Cg5 |
| *C. clementine vs C. ichangensis* | 22,288/1,512 | 15,497/372 | 13,088/207 | 9,362/88 | 14.74, 41.66, 63.23, 106.39 | 440 | Cc3-Ci3 |
| *C. clementine vs C. medica* | 20,146/1,479 | 13,447/349 | 10,985/177 | 7,511/66 | 13.62, 38.53, 62.06, 113.80 | 351 | Cc3-Cm3 |
| *C. clementine vs A. buxifolia* | 23,077/1,285 | 17,463/303 | 15,753/184 | 12,755/84 | 17.96, 57.63, 85.61, 151.85 | 796 | Cc5-Ab5 |
| *C. grandis vs C. ichangensis* | 23,434/1,587 | 16,490/418 | 13,753/231 | 9,208/87 | 14.77, 39.45, 59.54, 105.84 | 408 | Cg3-Ci3 |
| *C. grandis vs C. medica* | 21,259/1,560 | 14,293/390 | 11,611/203 | 7,429/69 | 13.63, 36.65, 57.20, 107.67 | 365 | Cg3-Cm3 |
| *C. grandis vs A. buxifolia* | 22,567/1,288 | 17,013/331 | 15,004/191 | 11,371/77 | 17.52, 51.40, 78.55, 147.68 | 796 | Cg5-Ab5 |
| *C. ichangensis vs C. medica* | 20,961/1,696 | 13,143/393 | 10,368/200 | 6,129/69 | 12.36, 33.44, 51.84, 88.83 | 221 | Ci7-Cm7 |
| *C. ichangensis vs A. buxifolia* | 20,693/1,488 | 14,193/392 | 11,558/210 | 7,177/74 | 13.91, 36.21, 55.04, 96.99 | 431 | Ci3-Ab3 |
| *C. medica vs A. buxifolia* | 20,032/1,551 | 12,949/366 | 10,189/176 | 6,820/70 | 12.92, 35.38, 57.89, 97.43 | 357 | Cm5-Ab5 |

BL: block length, ACGP: average collinear gene pairs respectively per block, LDB: number of collinear gene pairs reside in longest duplicated block

**Supplementary Table 4. Number of homologous blocks and genes within a genome or between genomes.**

| Homologous Blocks within and among genome | BL> 4 | BL > 10 | BL > 20 | BL > 50 | LDB | LDB on chromosomes |
| --- | --- | --- | --- | --- | --- | --- |
| *Vitis vinifera* | 3,666 | 2,578 | 1,872 | - | 46 | Vv1-Vv14/Vv4-Vv18 |
| *Citrus sinensis* | 3,792 | 2,109 | 1,222 | 258 | 67 | Cs1-Cs3 |
| *Citrus clementine* | 4,932 | 3,426 | 2,607 | 907 | 91 | Cc5-Cc9 |
| *Citrus grandis* | 4,750 | 2,558 | 1,689 | 456 | 106 | Cg5-Cg9 |
| *Citrus ichangensis* | 3,791 | 921 | 230 | - | 26 | Ci5-Ci7 |
| *Citrus medica* | 3,734 | 721 | 126 | - | 36 | Cm5-Cm9 |
| *Atalantia buxifolia* | 4,291 | 2,008 | 808 | 230 | 61 | Ab5-Ab7 |
| *V. vinifera vs C. sinensis* | 9,108/8,969 | 7,159/7,063 | 5,450/5,369 | 2,862/2,872 | 306 | Vv18-Cs3 |
| *V. vinifera vs C. clementine* | 10,313/10,473 | 8,722/8,733 | 7,154/7,095 | 4,284/4,308 | 317 | Vv18-Cc3 |
| *V. vinifera vs C. grandis* | 9,724/9,660 | 7,937/7,873 | 6,211/6,150 | 3,419/3,401 | 287 | Vv18-Cg3 |
| *V. vinifera vs C. ichangensis* | 8,697/8,615 | 5,878/5,727 | 3,575/3,514 | 1,045/1,042 | 209 | Vv18-Ci3 |
| *V. vinifera vs C. medica* | 8,092/8,010 | 5,206/5,132 | 2,999/2,973 | 824/824 | 210 | Vv18-Cm3 |
| *V. vinifera vs A. buxifolia* | 9438,/9,388 | 7,249/7,160 | 5,282/5,181 | 2,564/2,544 | 239 | Vv07-Ab5 |
| *C. sinensis vs C. clementine* | 13,786/14,177 | 12,858/13,081 | 11,849/11,994 | 10,006/10,018 | 1,328 | Cs3-Cc3 |
| *C. sinensis vs C. grandis* | 14,120/14,331 | 12,858/12,952 | 11,438/11,498 | 8,942/8,938 | 864 | Cs5-Cg5 |
| *C. sinensis vs C. ichangensis* | 11,663/11,798 | 9,850/9,873 | 8,162/8,164 | 5,082/5,093 | 437 | Cs1-Ci1 |
| *C. sinensis vs C. medica* | 10,342/10,563 | 8,645/8,638 | 7,346/7,354 | 5,207/5,219 | 388 | Cs7-Cm7 |
| *C. sinensis vs A. buxifolia* | 12,789/12,951 | 11,428/11,497 | 10,093/10,116 | 7,399/7,386 | 924 | Cs5-Ab5 |
| *C. clementine vs C. grandis* | 15,716/15,474 | 14,717/14,581 | 13,661/13,572 | 11,596/11,555 | 1,356 | Cc5-Cg5 |
| *C. clementine vs C. ichangensis* | 13,253/13,143 | 10,940/10,795 | 8,705/8,642 | 4,938/4,941 | 440 | Cc3-Ci3 |
| *C. clementine vs C. medica* | 11,751/11,632 | 9,399/9,264 | 7,313/7,280 | 4,195/4,210 | 351 | Cc3-Cm3 |
| *C. clementine vs A. buxifolia* | 14,382/14,179 | 12,944/12,828 | 11,409/11,347 | 8,417/8,392 | 796 | Cc5-Ab5 |
| *C. grandis vs C. ichangensis* | 14,135/14,250 | 11,546/11,467 | 8,949/8,918 | 4,839/4,853 | 408 | Cg3-Ci3 |
| *C. grandis vs C. medica* | 12,473/12,527 | 9,820/9,804 | 7,428/7,429 | 3,972/3,977 | 365 | Cg3-Cm3 |
| *C. grandis vs A. buxifolia* | 14,090/14,083 | 12,379/12,367 | 10,595/10,586 | 7,413/7,413 | 796 | Cg5-Ab5 |
| *C. ichangensis vs C. medica* | 12,021/11,950 | 8,813/8,827 | 6,266/6,270 | 2,668/2,671 | 221 | Ci7-Cm7 |
| *C. ichangensis vs A. buxifolia* | 12,266/12,249 | 9,614/9,712 | 7,189/7,249 | 3,465/3,472 | 431 | Ci3-Ab3 |
| *C. medica vs A. buxifolia* | 11,413/11,419 | 8,785/8,787 | 6,557/6,550 | 3,309/3,309 | 357 | Cm5-Ab5 |

BL: block length, LDB: number of collinear gene pairs reside in longest duplicated block

**Supplementary Table 9. Citrus and their relative gene retention rates with *Vitis vinifera* as reference genome.**

| ***Vitis vinifera*** | | | | ***Citrus sinensis*** | | | ***Citrus clementine*** | | | ***Citrus grandis*** | | | ***Citrus medica*** | | | ***Citrus ichangensis*** | | | ***Atalantia buxifolia*** | | |
| --- | --- | --- | --- | --- | --- | --- | --- | --- | --- | --- | --- | --- | --- | --- | --- | --- | --- | --- | --- | --- | --- |
| **Chr** | **genes** | **Out-**  **para1** | **Out-**  **para2** | **Orth** | **Out-**  **para1** | **Out-**  **par2** | **Orth** | **Out-**  **para1** | **Out-**  **para2** | **Orth** | **Out**  **para1** | **Out-**  **para2** | **Orth** | **Out-**  **para1** | **Out-**  **para2** | **Orth** | **Out-**  **para1** | **Out-**  **para2** | **Orth** | **Out-**  **par1** | **Out-**  **par2** |
| **1** | 1,406 | 0.144 | 0.157 | 0.652 | 0.105 | 0.100 | 0.847 | 0.110 | 0.113 | 0.784 | 0.104 | 0.111 | 0.369 | 0.076 | 0.063 | 0.466 | 0.068 | 0.06 | 0.43 | 0.086 | 0.091 |
| **2** | 976 | 0.134 | 0.110 | 0.830 | 0.068 | 0.006 | 0.787 | 0.104 | 0.078 | 0.726 | 0.076 | 0.082 | 0.355 | 0.054 | 0.006 | 0.596 | 0.042 | 0.004 | 0.679 | 0.048 | 0.006 |
| **3** | 1.132 | 0.160 | 0.118 | 0.622 | 0.120 | 0.063 | 0.877 | 0.125 | 0.077 | 0.735 | 0.117 | 0.063 | 0.355 | 0.087 | 0.047 | 0.215 | 0.105 | 0.038 | 0.365 | 0.108 | 0.002 |
| **4** | 1,368 | 0.151 | 0.126 | 0.788 | 0.115 | 0.080 | 0.698 | 0.116 | 0.100 | 0.687 | 0.120 | 0.086 | 0.35 | 0.09 | 0.048 | 0.633 | 0.074 | 0.035 | 0.437 | 0.105 | 0.04 |
| **5** | 1,445 | 0.125 | 0.070 | 0.662 | 0.080 | 0.036 | 0.792 | 0.100 | 0.046 | 0.827 | 0.096 | 0.036 | 0.396 | 0.06 | 0.02 | 0.411 | 0.046 | 0.019 | 0.536 | 0.074 | 0.029 |
| **6** | 1,289 | 0.240 | 0.157 | 0.729 | 0.153 | 0.079 | 0.843 | 0.192 | 0.094 | 0.77 | 0.167 | 0.099 | 0.38 | 0.097 | 0.031 | 0.492 | 0.118 | 0.041 | 0.522 | 0.098 | 0.071 |
| **7** | 1,430 | 0.225 | 0.090 | 0.707 | 0.167 | 0.048 | 0.768 | 0.180 | 0.055 | 0.789 | 0.171 | 0.059 | 0.36 | 0.11 | 0.035 | 0.52 | 0.12 | 0.023 | 0.478 | 0.151 | 0.036 |
| **8** | 1,488 | 0.198 | 0.132 | 0.614 | 0.123 | 0.081 | 0.860 | 0.143 | 0.092 | 0.764 | 0.128 | 0.093 | 0.36 | 0.062 | 0.034 | 0.446 | 0.08 | 0.054 | 0.432 | 0.091 | 0.077 |
| **9** | 1,140 | 0.131 | 0.134 | 0.656 | 0.084 | 0.092 | 0.698 | 0.080 | 0.096 | 0.679 | 0.07 | 0.088 | 0.407 | 0.03 | 0.062 | 0.333 | 0.082 | 0.058 | 0.301 | 0.08 | 0.07 |
| **10** | 894 | 0.108 | 0.064 | 0.774 | 0.062 | 0.038 | 0.792 | 0.070 | 0.044 | 0.736 | 0.06 | 0.042 | 0.22 | 0.022 | 0.018 | 0.478 | 0.042 | 0.028 | 0.728 | 0.03 | 0.032 |
| **11** | 1,093 | 0.127 | 0.132 | 0.766 | 0.086 | 0.102 | 0.828 | 0.096 | 0.090 | 0.744 | 0.081 | 0.072 | 0.552 | 0.063 | 0.038 | 0.446 | 0.043 | 0.098 | 0.638 | 0.038 | 0.092 |
| **12** | 1,299 | 0.092 | 0.090 | 0.650 | 0.055 | 0.062 | 0.796 | 0.063 | 0.065 | 0.752 | 0.063 | 0.053 | 0.284 | 0.028 | 0.018 | 0.357 | 0.033 | 0.018 | 0.45 | 0.052 | 0.053 |
| **13** | 1,437 | 0.190 | 0.183 | 0.610 | 0.125 | 0.092 | 0.732 | 0.130 | 0.134 | 0.721 | 0.133 | 0.120 | 0.281 | 0.059 | 0.056 | 0.397 | 0.095 | 0.053 | 0.524 | 0.093 | 0.049 |
| **14** | 1,625 | 0.216 | 0.19 | 0.744 | 0.152 | 0.118 | 0.800 | 0.170 | 0.150 | 0.768 | 0.16 | 0.145 | 0.499 | 0.102 | 0.074 | 0.507 | 0.118 | 0.086 | 0.645 | 0.114 | 0.105 |
| **15** | 957 | 0.168 | 0.146 | 0.226 | 0.137 | 0.072 | 0.843 | 0.140 | 0.096 | 0.826 | 0.117 | 0.082 | 0.064 | 0.076 | 0.05 | 0.178 | 0.115 | 0.041 | 0.195 | 0.12 | 0.058 |
| **16** | 1,076 | 0.154 | 0.130 | 0.566 | 0.117 | 0.014 | 0.817 | 0.117 | 0.092 | 0.624 | 0.115 | 0.092 | 0.314 | 0.035 | 0.014 | 0.306 | 0.097 | 0.01 | 0.44 | 0.107 | 0.012 |
| **17** | 1,021 | 0.231 | 0.189 | 0.622 | 0.167 | 0.118 | 0.874 | 0.199 | 0.128 | 0.755 | 0.185 | 0.117 | 0.441 | 0.088 | 0.083 | 0.392 | 0.113 | 0.074 | 0.535 | 0.112 | 0.097 |
| **18** | 2,007 | 0.136 | 0.095 | 0.820 | 0.084 | 0.066 | 0.860 | 0.082 | 0.081 | 0.843 | 0.081 | 0.071 | 0.611 | 0.053 | 0.04 | 0.608 | 0.055 | 0.021 | 0.759 | 0.022 | 0.039 |
| **19** | 1,200 | 0.097 | 0.053 | 0.634 | 0.060 | 0.032 | 0.805 | 0.072 | 0.040 | 0.833 | 0.072 | 0.035 | 0.337 | 0.02 | 0.01 | 0.337 | 0.028 | 0.008 | 0.531 | 0.04 | 0.027 |

**Supplementary Table 10. Citrus and their relative gene retention rates with *Citrus sinensis* as reference genome.**

| ***Citrus sinenis*** | | | | ***Citrus clementine*** | | | ***Citrus grandis*** | | | ***Citrus medica*** | | | ***Citrus ichangensis*** | | | ***Atalantia buxifolia*** | | |
| --- | --- | --- | --- | --- | --- | --- | --- | --- | --- | --- | --- | --- | --- | --- | --- | --- | --- | --- |
| **Chr** | **Genes** | **para 1** | **para 2** | **Orth** | **Out-**  **para 1** | **Out-**  **para 2** | **Orth** | **Out-**  **para 1** | **Out-**  **para 2** | **Orth** | **Out-**  **para 1** | **Out-**  **para 2** | **Orth** | **Out-**  **para 1** | **Out-**  **para 2** | **Orth** | **Out-**  **para 1** | **Out-**  **para 2** |
| 1 | 2,690 | 0.209 | 0.029 | 0.798 | 0.187 | 0.027 | 0.800 | 0.176 | 0.028 | 0.561 | 0.156 | 0.021 | 0.618 | 0.172 | 0.026 | 0.683 | 0.173 | 0.024 |
| 2 | 3,136 | 0.201 | 0.033 | 0.790 | 0.173 | 0.028 | 0.818 | 0.170 | 0.029 | 0.522 | 0.133 | 0.023 | 0.700 | 0.144 | 0.025 | 0.725 | 0.155 | 0.028 |
| 3 | 2,799 | 0.186 | 0.032 | 0.769 | 0.158 | 0.029 | 0.800 | 0.156 | 0.030 | 0.618 | 0.129 | 0.026 | 0.679 | 0.140 | 0.026 | 0.725 | 0.154 | 0.026 |
| 4 | 2,009 | 0.193 | 0.024 | 0.786 | 0.167 | 0.020 | 0.809 | 0.170 | 0.022 | 0.621 | 0.130 | 0.019 | 0.684 | 0.131 | 0.020 | 0.796 | 0.159 | 0.017 |
| 5 | 3,554 | 0.174 | 0.023 | 0.788 | 0.157 | 0.021 | 0.842 | 0.152 | 0.021 | 0.593 | 0.116 | 0.018 | 0.639 | 0.134 | 0.021 | 0.714 | 0.148 | 0.020 |
| 6 | 2,200 | 0.212 | 0.036 | 0.826 | 0.190 | 0.033 | 0.800 | 0.189 | 0.034 | 0.676 | 0.144 | 0.024 | 0.766 | 0.171 | 0.029 | 0.708 | 0.175 | 0.031 |
| 7 | 3,229 | 0.214 | 0.034 | 0.809 | 0.195 | 0.031 | 0.819 | 0.189 | 0.031 | 0.539 | 0.155 | 0.022 | 0.706 | 0.175 | 0.026 | 0.765 | 0.184 | 0.028 |
| 8 | 2,067 | 0.232 | 0.033 | 0.760 | 0.199 | 0.028 | 0.803 | 0.204 | 0.029 | 0.555 | 0.149 | 0.023 | 0.606 | 0.174 | 0.027 | 0.616 | 0.184 | 0.026 |
| 9 | 1,898 | 0.163 | 0.017 | 0.768 | 0.147 | 0.016 | 0.786 | 0.143 | 0.015 | 0.600 | 0.111 | 0.012 | 0.621 | 0.131 | 0.015 | 0.756 | 0.134 | 0.015 |

**Supplementary Table 11. The observed distribution of gene loss and translocation numbers fitted by using different density curves of geometry distribution.**

| **Reference genome** | **Genomes** | **Parameter of geometry distribution** | **Fitness (R-square)** | **P-value (F-test)** |
| --- | --- | --- | --- | --- |
| ***Vitis Vinifera*** | ***Citrus sinensis*** | 0.5344 | 0.9947 | 0.9208 |
|  | ***Citrus clementine*** | 0.5396 | 0.997 | 0.9363 |
|  | ***Citrus grandis*** | 0.5230 | 0.9971 | 0.9356 |
|  | ***Citrus ichangensis*** | 0.4676 | 0.9961 | 0.9091 |
|  | ***Citrus medica*** | 0.4781 | 0.9921 | 0.8852 |
|  | ***Atalantia buxifolia*** | 0.4977 | 0.9943 | 0.9219 |
| ***Citrus sinensis*** | ***Citrus clementine*** | 0.6470 | 0.9975 | 0.9557 |
|  | ***Citrus grandis*** | 0.6661 | 0.9986 | 0.9635 |
|  | ***Citrus ichangensis*** | 0.6288 | 0.9941 | 0.9274 |
|  | ***Citrus medica*** | 0.5991 | 0.9908 | 0.9093 |
|  | ***Atalantia buxifolia*** | 0.6341 | 0.9965 | 0.9507 |

**Supplementary Table 12. Kernel function analysis of Ks distribution related to duplication events within each genome and between selected Rutaceae plants (before evolutionary rate correction).**

| **Genomes** | **Weight coefficient related to ECH or speciation** | **Peak of Ks distribution (μ1) related to ECH or speciation** | **Deviation (σ1)** |
| --- | --- | --- | --- |
| ***V. vinifera*** | 0.926 | 1.053 | 0.293 |
| ***C. sinensis*** | 0.900 | 1.301 | 0.343 |
| ***C. clementina*** | 0.949 | 1.295 | 0.387 |
| ***C. grandis*** | 0.916 | 1.288 | 0.419 |
| ***C. ichangensis*** | 0.920 | 1.252 | 0.343 |
| ***C. medica*** | 0.951 | 1.256 | 0.326 |
| ***A. buxifolia*** | 0.969 | 1.297 | 0.414 |
| ***V. Vinifera vs C. sinensis*** | 0.895 | 0.831 | 0.233 |
| ***C. Sinensis vs A. buxifolia*** | 0.477 | 0.063 | 0.060 |
| ***C. Sinensis vs C. medica*** | 0.475 | 0.025 | 0.062 |
| ***C. Sinensis vs C. clementina*** | 0.463 | 0.011 | 0.060 |
| ***C. Grandis vs C. ichangensis*** | 0.527 | 0.018 | 0.063 |

**Supplementary Table 13. Kernel function analysis of Ks distribution related to duplication events within each genome and between selected Rutaceae plants (after evolutionary rate correction).**

| **Genomes** | **Weight coefficient related to ECH or speciation** | **Peak of Ks distribution (μ1) related to ECH or speciation** | **Deviation (σ1)** | **Correction coefficient (λ)** |
| --- | --- | --- | --- | --- |
| ***V. vinifera*** | 0.926 | 1.053 | 0.293 | 1 |
| ***C. sinensis*** | 0.926 | 1.053 | 0.296 | 0.809 |
| ***C. clementina*** | 0.950 | 1.053 | 0.317 | 0.813 |
| ***C. grandis*** | 0.918 | 1.053 | 0.345 | 0.818 |
| ***C. ichangensis*** | 0.901 | 1.053 | 0.291 | 0.841 |
| ***C. medica*** | 0.948 | 1.053 | 0.359 | 0.838 |
| ***A. buxifolia*** | 0.970 | 1.053 | 0.338 | 0.812 |
| ***V. Vinifera vs C. sinensis*** | 0.897 | 0.744 | 0.210 | 0.895 |
| ***C. Sinensis vs A. buxifolia*** | 0.451 | 0.052 | 0.059 | 0.811 |
| ***C. Sinensis vs C. medica*** | 0.503 | 0.020 | 0.062 | 0.824 |
| ***C. Sinensis vs C. clementina*** | 0.573 | 0.010 | 0.058 | 0.811 |
| ***C. Grandis vs C. ichangensis*** | 0.542 | 0.016 | 0.061 | 0.829 |

**Supplementary Table 14. Vitamin C genes related to duplication event in each Rutaceae genome.**

| **Subfamily**  **ECH-related genes (collinear regions)/Total genes** | ***Vitis***  ***vinifera*** | ***Citrus***  ***sinensis*** | ***Citrus***  ***clementine*** | ***Citrus***  ***grandis*** | ***Citrus***  ***medica*** | ***Citrus***  ***ichangensis*** | ***Atalantia***  ***buxifolia*** |
| --- | --- | --- | --- | --- | --- | --- | --- |
| **Putative nucleobase-ascorbate transporter (AAT)** | 6/7 | 6/7 | 6/15 | 6/7 | 1/6 | 3/9 | 6/10 |
| **ATPase family AAA domain-containing protein (Alase)** | 1/2 | 1/2 | 1/3 | 1/2 | 1/2 | 1/2 | 1/2 |
| **L-ascorbate oxidase (AO)** | 2/7 | 1/2 | 2/2 | 2/3 | 0/4 | 1/4 | 2/2 |
| **Putative L-ascorbate peroxidase (APX)** | 5/6 | 4/5 | 4/5 | 4/5 | 4/5 | 4/6 | 4/6 |
| **Putative glutathione S-transferase (DHAR)** | 1/2 | 2/2 | 2/3 | 2/2 | 1/2 | 1/2 | 2/2 |
| **NAD(P)H-dependent oxidoreductase 1; Aldo-keto reductase family member (GalUR)** | 5/26 | 4/9 | 5/28 | 5/17 | 2/24 | 2/18 | 6/14 |
| **2,5-diketo-D-gluconic acid reductase (GalDH)** | 0/1 | 1/1 | 1/1 | 1/1 | 0/1 | 0/1 | 1/1 |
| **Putative GDP-L-fucose synthase (GER)** | 0/1 | 0/1 | 1/1 | 0/1 | 0/1 | 0/1 | 1/1 |
| **GDP-L-galactose phosphorylase (GGalPP)** | 2/3 | 2/2 | 2/3 | 2/3 | 1/3 | 2/2 | 2/2 |
| **L-galactono-1,4-lactone dehydrogenase (GalLDH)** | 1/1 | 0/2 | 0/1 | 0/1 | 0/1 | 0/1 | 0/1 |
| **Probable GDP-mannose 4,6-dehydratase (GMD)** | 1/1 | 1/1 | 1/1 | 1/1 | 1/2 | 1/4 | 0/1 |
| **GDP-mannose 3,5-epimerase (GME)** | 2/2 | 1/3 | 2/2 | 2/2 | 1/2 | 0/4 | 1/3 |
| **Probable mannose-1-phosphate guanylyltransferase (GMPase)** | 1/1 | 1/2 | 2/3 | 2/2 | 0/2 | 1/2 | 2/3 |
| **Probable mannose-1-phosphate guanylyltransferase (GalPP)** | 4/5 | 0/1 | 2/6 | 2/4 | 0/5 | 0/7 | 3/6 |
| **L-gulono-1,4-lactone oxidase (GLOase)** | 1/1 | 0/2 | 0/1 | 0/1 | 0/1 | 0/1 | 0/1 |
| **Probable inositol-3-phosphate synthase isozyme (IPS)** | 1/1 | 1/1 | 1/2 | 1/1 | 0/1 | 1/1 | 1/1 |
| **Probable monodehydroascorbate reductase (MDAR)** | 3/3 | 3/3 | 3/9 | 3/3 | 2/3 | 2/3 | 3/3 |
| **Probable inositol oxygenase (MIOX)** | 2/3 | 2/2 | 2/7 | 2/4 | 3/4 | 0/5 | 2/4 |
| **Probable polygalacturonase; Endo-polygalacturonase (PG)** | 7/11 | 5/9 | 5/9 | 5/8 | 3/15 | 4/10 | 4/8 |
| **Glucose-6-phosphate isomerase (PGI)** | 1/2 | 2/2 | 2/3 | 2/2 | 1/4 | 1/3 | 2/2 |
| **Putative pectinesterase/pectinesterase inhibitor (PME)** | 14/29 | 7/29 | 10/35 | 6/32 | 5/22 | 5/38 | 9/29 |
| **Probable mannose-6-phosphate isomerase; Mannose-6-phosphate isomerase (PMI)** | 2/2 | 2/2 | 2/2 | 2/2 | 0/2 | 2/2 | 2/2 |
| **Probable phosphomannomutase (PMM)** | 1/1 | 0/1 | 1/1 | 1/1 | 0/1 | 0/1 | 0/1 |
| **Total genes (All genes in collinear regions)** | 63/118 | 46/101 | 57/143 | 52/105 | 26/113 | 31/127 | 54/105 |
| **Percent collinear genes in whole genome** | 53.39% | 45.54% | 39.86% | 49.52% | 23.01% | 24.41% | 51.43% |

**References**

Jaillon, O., Aury, J.M., Noel, B., Policriti, A., Clepet, C., Casagrande, A., et al. (2007). The grapevine genome sequence suggests ancestral hexaploidization in major angiosperm phyla. *Nature* 449(7161)**,** 463-467. doi: 10.1038/nature06148.

Wang, X., Xu, Y., Zhang, S., Cao, L., Huang, Y., Cheng, J., et al. (2017). Genomic analyses of primitive, wild and cultivated citrus provide insights into asexual reproduction. *Nat Genet* 49(5)**,** 765-772. doi: 10.1038/ng.3839.

Wu, G.A., Prochnik, S., Jenkins, J., Salse, J., Hellsten, U., Murat, F., et al. (2014). Sequencing of diverse mandarin, pummelo and orange genomes reveals complex history of admixture during citrus domestication. *Nat Biotechnol* 32(7)**,** 656-662. doi: 10.1038/nbt.2906.

Xu, Q., Chen, L.L., Ruan, X., Chen, D., Zhu, A., Chen, C., et al. (2013). The draft genome of sweet orange (Citrus sinensis). *Nat Genet* 45(1)**,** 59-66. doi: 10.1038/ng.2472.
